# Coevolution of the bacterial pheromone ComS and sensor ComR fine-tunes natural transformation in streptococci

**DOI:** 10.1016/j.jbc.2021.101346

**Published:** 2021-10-27

**Authors:** Laura Ledesma-García, Imke Ensinck, Denis Dereinne, Felipe Viela, Johann Mignolet, Yves F. Dufrêne, Patrice Soumillion, Sylvie Nessler, Pascal Hols

**Affiliations:** 1 Louvain Institute of Biomolecular Science and Technology (LIBST), Université catholique de Louvain, Louvain-La-Neuve, Belgium; 2 CEA, CNRS, Institute for Integrative Biology of the Cell (I2BC), Université Paris-Saclay, Gif-sur-Yvette, France

**Keywords:** *streptococcus*, quorum sensing, cell signaling, DNA transformation, pheromone, cell-to-cell communication, competence, (R)RNPP, XIP, AFM, atomic force microscopy, CAP helix, capping helix α16, ComR_Sth_, *Streptococcus thermophilus* ComR, ComR_Sve_, *Streptococcus vestibularis* ComR, CSP, competence-stimulating peptide, FP, fluorescence polarization, HTH, helix-turn-helix, P_*comS*_, *comS* promoter, RLU, relative light unit, (R)RNPP, (Rgg,) Rap, NprR, PlcR, and PrgX, TPR, tetratricopeptide repeat, XIP, SigX-inducing peptide, XIP_Sth_, *Streptococcus thermophilus* XIP, XIP_Sve_, *Streptococcus vestibularis* XIP

## Abstract

Competence for natural transformation extensively contributes to genome evolution and the rapid adaptability of bacteria dwelling in challenging environments. In most streptococci, this process is tightly controlled by the ComRS signaling system, which is activated through the direct interaction between the (R)RNPP-type ComR sensor and XIP pheromone (mature ComS). The overall mechanism of activation and the basis of pheromone selectivity have been previously reported in Gram-positive salivarius streptococci; however, detailed 3D-remodeling of ComR leading up to its activation remains only partially understood. Here, we identified using a semirational mutagenesis approach two residues in the pheromone XIP that bolster ComR sensor activation by interacting with two aromatic residues of its XIP-binding pocket. Random and targeted mutagenesis of ComR revealed that the interplay between these four residues remodels a network of aromatic–aromatic interactions involved in relaxing the sequestration of the DNA-binding domain. Based on these data, we propose a comprehensive model for ComR activation based on two major conformational changes of the XIP-binding domain. Notably, the stimulation of this newly identified trigger point by a single XIP substitution resulted in higher competence and enhanced transformability, suggesting that pheromone-sensor coevolution counter-selects for hyperactive systems in order to maintain a trade-off between competence and bacterial fitness. Overall, this study sheds new light on the ComRS activation mechanism and how it could be exploited for biotechnological and biomedical purposes.

Bacteria have developed diverse horizontal gene transfer mechanisms that favor their adaptation and survival to a fluctuating and competitive ecological niche (1). Among those, natural DNA transformation allows the acquisition of new genetic traits from phylogenetically close and distant species (2, 3, 4). Regarding pathogenic bacteria, this increase in genome plasticity has been linked to the gain of virulence-related mechanisms and multidrug resistance, improving their success during host infection (2, 5).

During the natural transformation process, bacteria must enter a transitory physiological state called competence (3, 6), where a master transcriptional regulator triggers the expression of the genes encoding the transformasome (7). This multiprotein machinery captures, translocates, and finally integrates extracellular DNA fragments into the genome of competent bacteria (7). Competence is an energy-consuming process that results in fitness burden and affects cell division and chromosome integrity (8, 9). Hence, bacteria have orchestrated complex regulatory mechanisms to minimize the activation time window and avoid its fortuitous upregulation under inappropriate environmental conditions (10, 11, 12).

In streptococci, regulation of competence is primarily performed by the alternative sigma factor ComX (also called SigX, σ^X^) that transiently associates with the RNA polymerase to induce transcriptional reprogramming (13, 14). Besides, the proximal transcriptional control of this master regulator is executed by a cell-to-cell communication system based on short linear peptide pheromones (13). Two exclusive systems (*i.e.*, ComCDE or ComRS) trigger ComX production and ensure a robust competence activation through the boost of signaling peptide production (positive feedback loop) above a specific pheromone concentration threshold (13). Species of the mitis and anginosus groups use the Competence-Stimulating Peptide (CSP, mature form of the precursor ComC) to extracellularly stimulate the transmembrane histidine kinase ComD that autophosphorylates and subsequently activates the response regulator ComE, *via* a phosphorelay event (15, 16, 17). In contrast, in the salivarius, pyogenic, bovis, and suis groups, the SigX-Inducing Peptide (XIP, mature form of the precursor ComS) exported by producer cells is reimported into the intracellular space of responder cells to bind and activate the cytoplasmic transcriptional regulator ComR (Fig. 1*A*) (13, 18, 19, 20, 21, 22). Thus, the phosphorylated ComE or the complex ComR·XIP will bind specific promoter sequences to activate transcription of the competence regulon, including *comX* and their cognate pheromone-encoding gene (Fig. 1*A*) (13).

**Figure 1:**
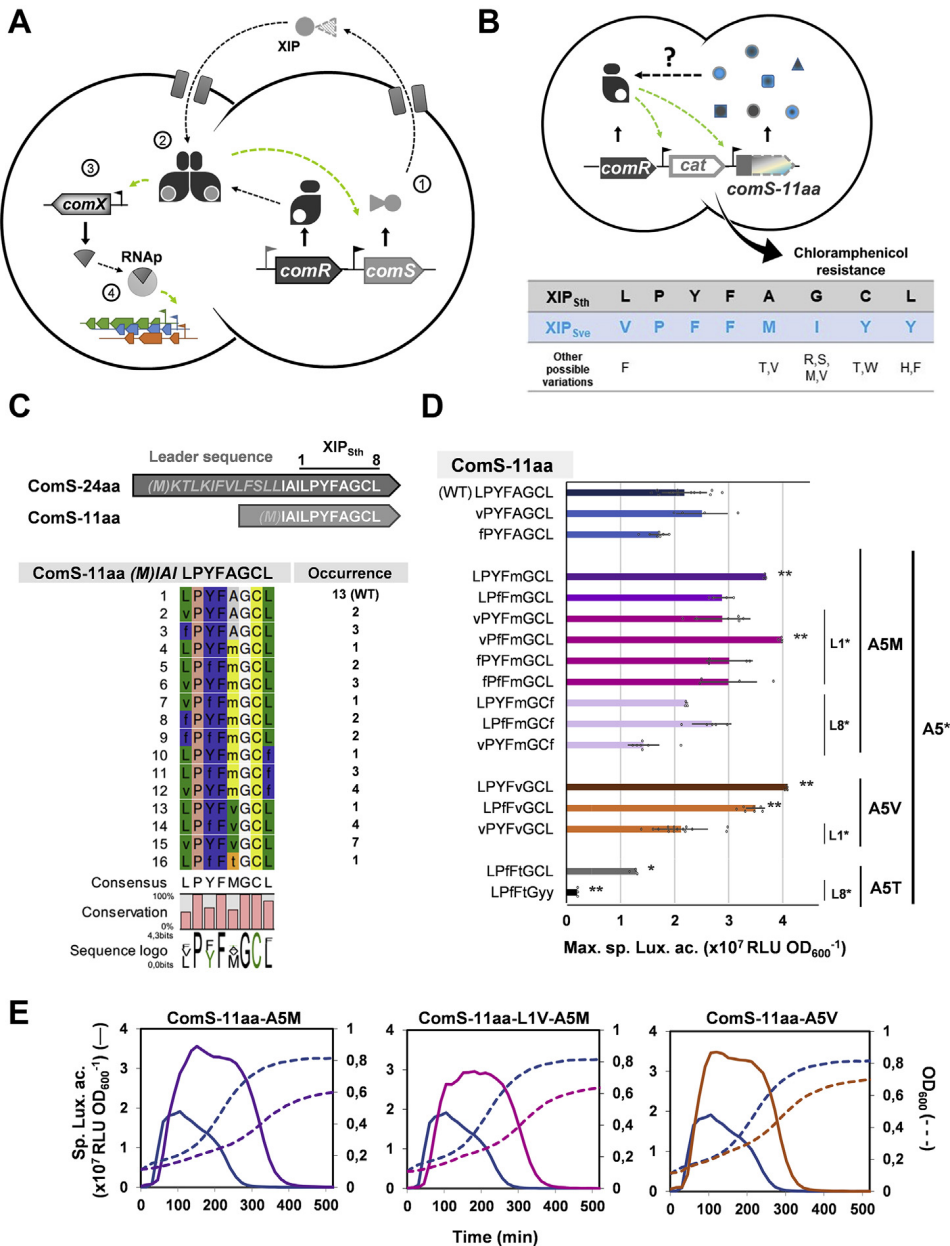
**XIP semirational mutagenesis.***A*, ComRS system in streptococci. The ComS precursor is exported and matured into the extracellular XIP peptide (1), (re)imported by an oligopeptide transporter for its interaction with the ComR sensor, triggering its dimerization (2). The resulting ComR·XIP complex activates *comS*, (positive feed-back loop) and *comX* (master regulator) (3). ComX (σ^X^) binds RNA-polymerase (RNAp) inducing competence transcriptional reprogramming (4). *B*, screening strategy for XIP mutagenesis. A library of semirandomized *comS-11aa* (*blue-gray arrow*, C-terminal 8 aa) under the control of the competence-inducible promoter (P_*comS*_) is expressed into a *S. thermophilus* reporter strain carrying a chloramphenicol resistance gene (*cat*) fused to P_*comS*_. ComR-activating peptides trigger their own production and chloramphenicol resistance. Residues corresponding to XIP_Sth_ (*gray*) and XIP_Sve_ (*blue*) were allowed in the degenerate oligonucleotide used for building the genetic library. Other possible residues at each XIP position are indicated below. *C*, sequence alignment of ComR_Sth_-activating peptide variants. The sequences of the full-length precursor (ComS-24aa, leader sequence in *gray*), the native extracellular XIP peptide (ComS-11aa, shown in *white*), and the minimal interacting XIP peptide (XIP_Sth_, 8 aa at C-terminus) of *S. thermophilus* are indicated on the *top* of the alignment. The occurrence of each peptide variant among 51 selected clones is indicated on the *right*. Residues are color-coded according to the Rasmol color scheme (53) and the consensus sequence is shown underneath. The Bits represent the relative frequency of residues. *D*, maximum specific luciferase activity (RLU OD_600_^−1^) of cytoplasmically expressed peptide variants. Activation by ComS-11aa (M)IAILPYFAGCL (positive control), (M)IAILPfFtGyy variant (negative control) or other peptide variants was monitored from a strain carrying a P_*comS*_-*luxAB* fusion. Peptide variants carrying methionine, valine, or threonine substitutions at XIP position 5 are colored in *purple*, *orange*, or *gray* spectra, respectively. ∗ indicates a mutation at residue L1, A5, or L8. Experimental values are mean ± SD of at least three independent replicates. Significant differences (ComS-11aa WT as reference) calculated by Student unpaired *t* test are indicated (∗∗*p*-value < 0.01, ∗*p*-value < 0.05). *E*, kinetics of luciferase activation (*solid lines*) and growth curves (*dotted lines*) of reporter strains producing peptide variants ComS-11aa-A5M (*purple*), ComS-11aa-L1V-A5M (*fuchsia*), or ComS-11aa-A5V (*orange*). The control ComS-11aa WT (*blue*) is represented in all graphs. One representative experiment of three independent experiments showing similar results.

ComR belongs to the (R)RNPP (for the original members (Rgg,) Rap, NprR, PlcR, and PrgX) superfamily of transcriptional regulators ubiquitous in Firmicutes (23). Several crystal structures of (R)RNPPs have been solved, demonstrating that they canonically exhibit an all-α two-domain structure formed by a N-terminal helix-turn-helix (HTH) DNA-binding domain and a C-terminal tetratricopeptide repeat (TPR)-type peptide-binding domain (23, 24, 25, 26, 27, 28). In the case of ComR, the HTH-domain is composed of five α-helices while the TPR-domain encompasses five pairs of anti-parallel α-helices (forming 5 TPR-subdomains) and a single C-terminal CAP helix (capping helix α16) (27, 29). These detailed structure–function studies revealed that all (R)RNPPs undergo various conformational changes upon pheromone interaction that result in very diverse regulatory mechanisms (24, 26). In contrast to other members of the family, ComR is monomeric in the absence of peptide and only adopts its active dimeric form in the presence of XIP (29, 30). While apo-ComR displays a sequestered HTH domain fastened by the TPR domain, XIP binding induces conformational changes resulting in dimerization of the TPR domain and allosteric release of the two DNA-binding domains (29, 30). This idiosyncratic molecular mechanism ensures locking of the transcriptional regulator in the absence of its specific inducing pheromone.

Phylogenetic studies suggested a pheromone-sensor coevolution, which leads to primary sequence divergences among ComRS systems, often resulting in the absence of cross talk between streptococcal species (19, 27). Structural comparison between ComR·XIP complexes from two species of the salivarius group that do not cross talk, *Streptococcus thermophilus* and *Streptococcus vestibularis*, showed a conserved XIP-binding mode and helped pinpointing the minimal key residues in ComR and XIP required for peptide selectivity (30). These studies also allowed us to refine the activation model of the ComRS system. Main pillars of the TPR conformational change triggering dimerization and release of the HTH domain were highlighted. In particular, we showed the essential role of the XIP-induced reorientation of the TPR-1 (α6 and α7 helices) and TPR-2 (α8 and α9 helices) subdomains and recruitment of the CAP helix. In addition, these studies also suggested that additional interactions, such as those observed between two ComR aromatic residues and the hydrophobic residue at XIP N-terminus, could also play a major role in the activation mechanism and peptide selectivity (29, 30).

In this work, we use accelerated evolution of XIP pheromones from two orthologous systems to shed light on novel aspects of ComRS evolution and activation mechanism. Notably, a single pheromone mutation that increases ComR-XIP hydrophobic contacts drastically enhances DNA transformation, suggesting that evolution has kept this process under strict control to minimize fitness costs. A detailed reciprocal investigation of ComR-XIP interacting residues revealed an underestimated critical step of the ComRS activation mechanism.

## Results

### Exploring the landscape of active peptides between salivarius streptococci pheromones

In order to identify peptide variants that could foster ComR activation in *S. thermophilus*, an *in vivo* method based on their positive screening in selective medium was designed by fusing a ComR-responsive promoter (*comS* promoter [P_*comS*_] as proxy) to a gene conferring resistance to chloramphenicol (P_*comS*_*-cat*). Then, we generated a small semirational peptide library by using degenerate primers allowing all residue combinations between XIP_Sth_ (L_1_PYFAGCL_8_) from *S. thermophilus* and XIP_Sve_ (V_1_PFFMIYY_8_) from *S. vestibularis* (Fig. 1*B*) (30). Due to the degenerate nature of the genetic code, residues not present in XIP_Sth_ and XIP_Sve_ sequences were possible at some positions (Fig. 1*B*), resulting in a predicted library of 2304 peptide variants. To minimize interferences with degradation and translocation processes, we chose to express peptide variants with three additional N-terminal residues corresponding to the native extracellular peptide (ComS_Δ2–13_, 11 aa) (20). These peptides are unable to be exported in the spontaneously transformable strain *S. thermophilus* LMD-9. The system was validated for competence activation using a luminescence reporter strain (P_*comS*_-*luxAB*) (Fig. S1*A*).

A total of 51 candidates were identified on chloramphenicol plates with the most prevalent peptide (13 instances) corresponding to wild-type XIP_Sth_. The alignment of 15 nonredundant peptide variants with the reference sequence (Fig. 1*C*) showed that most of the permissive positions (four out of six) could be mutated. The absence of variations at two positions, XIP_Sth_ G6 and C7, strikingly suggests that their modifications can drastically affect peptide·ComR complex formation/activation. XIP_Sth_ position 3 was occupied by either a tyrosine (∼60% of peptide variants) or a phenylalanine (∼40%), supporting previous results that a substitution at this position by an alternative aromatic amino acid is tolerated without major effect (19). Interestingly, ∼20% of peptide variants showed that XIP_Sth_ L8, of which carboxylate group was reported as critical for ComR binding (29, 30), could be replaced by the aromatic residue phenylalanine when present with the additional A5M mutation. Finally, XIP_Sth_ L1 and A5 showed the highest permissiveness by displaying all allowed modifications. Substitutions at those two positions are found in 50% and ∼80% of peptide variants, respectively. The permissiveness of these two XIP positions correlates with natural variations observed in XIPs from salivarius streptococci, as XIP-1 and XIP-5 can be substituted by L, V, A, C and A, T, M, I, respectively (30).

Altogether, this semirational mutagenesis supports the high permissiveness of ComR_Sth_ for its activation by a range of XIP variants (30). However, the observed tolerability at each XIP position is strikingly variable: from strict (*i.e.*, XIP-6 and XIP-7) to highly permissive (*i.e.*, XIP-1 and XIP-5) with a predominant tolerance at XIP position 5.

### Cytoplasmic production of XIP-5 variants improves ComR_Sth_ activation

The capacity of the peptide variants for transcriptional activation was measured by luminescence assays (P_*comS*_ as proxy) and compared with the strain producing ComS_Sth_-11 aa (positive control) (Fig. 1*D*). A potential candidate (LPFFTGYY at C-terminus) that was unable to grow on selective medium was also incorporated as negative control (Figs. 1*D* and S1*B*).

Peptide variants carrying a single modification in L1 (L1V or L1F) were not significantly altered in their activation capacity while those harboring A5M or A5V increased up to twice the signal compared with the wild-type sequence. Moreover, strains carrying those last two modifications displayed an extended activation accompanied by a slight decrease of growth as expected for a higher competence triggering (Fig. 1*E*) (18, 31). In most cases, the association of A5M or A5V with single or multiple L1/Y3/L8 modifications reduced the activation capacity (Fig. 1*D*). The most severe reduction included a combination of A5M with L8F, supporting previous data on the importance of a branched-chain amino acid at XIP-8 for optimal ComR activation (19).

This expression analysis suggests that modifications at XIP position 5 (*i.e.*, A5M or A5V) boosts ComR activation and may indicate that the native XIP_Sth_ is not the most active pheromone variant.

### Exogenous supply and native production of XIP-A5M increase competence

**Figure 2:**
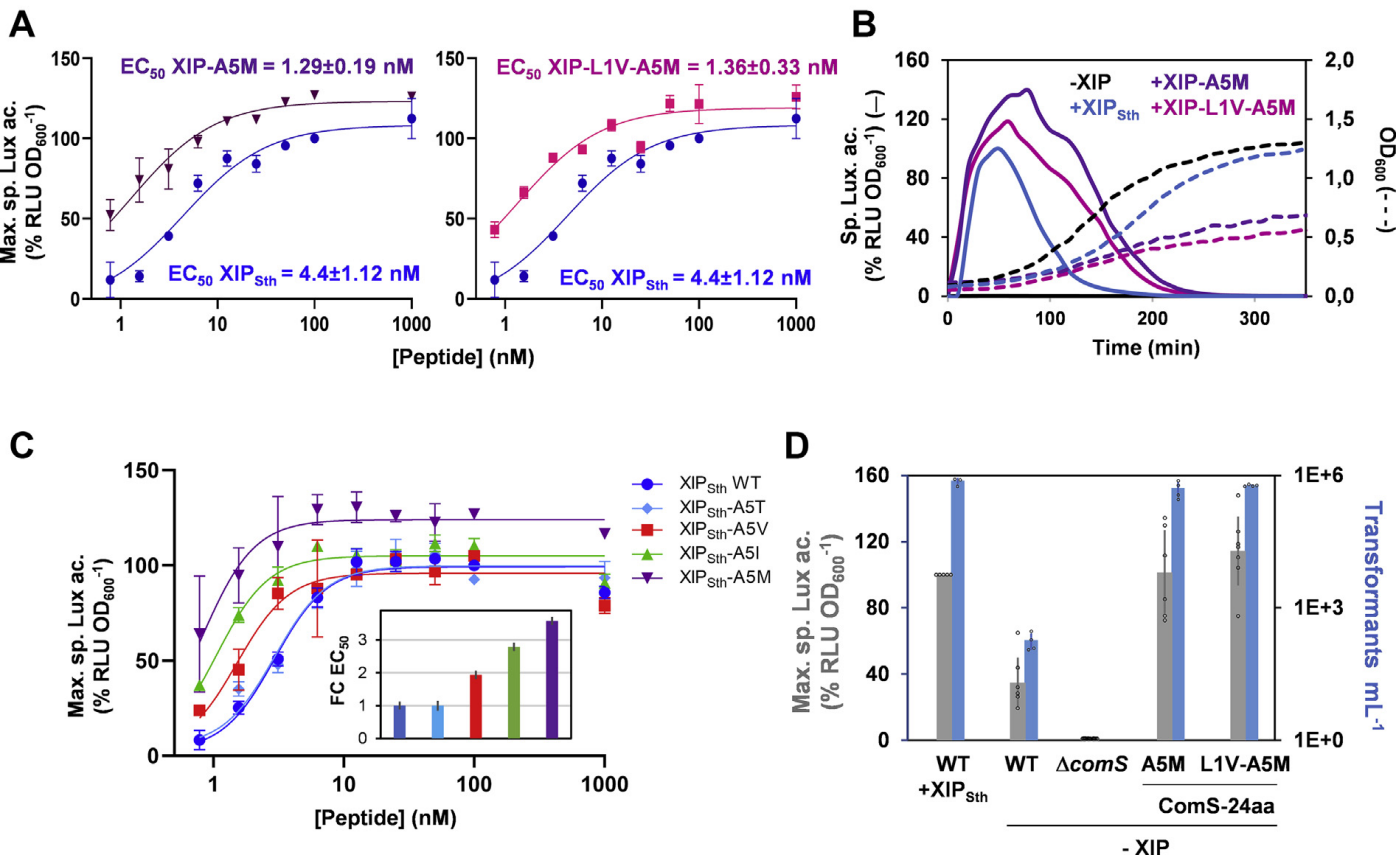
***In vivo* effect of synthetic pheromone variants.***A*, dose response of P_*comS*_ activity upon addition of synthetic (8 aa) XIP_Sth_ WT (*blue*), XIP-A5M (*purple*), or XIP-L1V-A5M (*fuchsia*). Maximum specific luciferase activity (% RLU × OD_600_^−1^) was monitored by using a *S. thermophilus* Δ*comS* reporter strain (P_*comS*_-*luxAB*). The value obtained in the presence of 100 nM XIP_Sth_ was used as reference. Plots were fitted with the Hill equation to calculate the EC_50_ values that are indicated in the graphs. *B*, kinetics of luciferase activation (*solid lines*) and growth curves (*dotted lines*) of the reporter strain upon addition (100 nM) of synthetic XIP_Sth_ WT (*blue*), XIP-A5M (*purple*), or XIP-L1V-A5M (*fuchsia*), or in absence of peptide (*black*). *C*, dose response of P_*comS*_ activity upon addition of synthetic XIP_Sth_ WT (*blue*), or XIP-5 variants: XIP-A5T (*light blue*), XIP-A5V (*red*), XIP-A5I (*green*), or XIP-A5M (*purple*). The monitoring of luciferase activity and estimation of EC_50_ were performed as in panel *A*. The *inset* represents the fold change (FC) in EC_50_ of each XIP-5 variant using as reference the EC_50_ of XIP_Sth_ WT. Increase in fold change is representative of a decrease in EC_50_ compared with XIP_Sth_ WT fixed at 1. *D*, maximum specific luciferase activity (% RLU × OD_600_^−1^; *gray bars*) and transformation efficiency (transformants × ml^−1^; *blue bars*) of *S. thermophilus* reporter strains producing the full-length precursor ComS-24aa WT, ComS-24aa-A5M or ComS-24aa-L1V-A5M. *S. thermophilus* WT in the presence of 100 nM XIP_Sth_ and a Δ*comS* mutant strain were included as positive and negative control, respectively. The WT strain in the presence of XIP_Sth_ is used as reference to normalize the maximum specific luciferase activity. In *A*, *C*, and *D*, experimental values represent the mean ± SD of at least three independent replicates.

In order to confirm the data obtained from the screening, the last 8 aa of two more active peptides (*i.e.*, XIP-A5M and XIP-L1V-A5M) were chemically synthetized. These peptide variants were selected in order to study the effect of the A5M variation alone or in combination with a modification at XIP position 1. Luminescence assays with increasing extracellular concentrations of those peptides were performed using a reporter strain (P_*comS*_ as proxy) lacking the *comS* gene (Fig. 2*A*). In agreement with screening data, addition of XIP-A5M and XIP-L1V-A5M resulted in a similar increased and extended transcriptional activation with an approximately fourfold lower EC_50_ than wild-type XIP_Sth_ (Fig. 2, *A* and *B*). As expected for a strong competence activation, the addition of these two peptides had a negative effect on the growth rate of the reporter strain (Fig. 2*B*).

To investigate more deeply the role of substitutions at XIP position 5, we compared the impact of variations identified during the screen (*i.e.*, A5M, A5V, and A5T) or observed in some natural XIPs from salivarius streptococci (*i.e.*, A5T and A5I) (13, 30). The titration of the synthetic XIP_Sth_ variants showed that the A5T substitution is neutral while all the others are promoting activation (Fig. 2*C*). Considering the tested peptides, these results highlight a positive correlation between hydrophobicity (and bulkiness/length) of the side chain of XIP-5 residue and its ability to improve activation (A5M > A5I > A5V > A5T/A5). They also show that a methionine at that position is the most efficient to boost activation (Fig. 2*C*).

Finally, we evaluated if the genome-encoded version of XIP-A5M or and XIP-L1V-A5M as full-length precursor (24 aa) could be able to spontaneously activate DNA transformation. Reporter strains harboring the full-length *comS* gene encoding the peptide variants were constructed by site-directed mutagenesis. Then, their capacity for transcriptional activation (P_*comS*_ as proxy) and spontaneous transformation were measured (Fig. 2*D*). A direct correlation between an enhanced competence gene expression and an increased transformation yield was confirmed for the native expression of XIP-A5M and XIP-L1V-A5M. Remarkably, the transformation rate of these two mutants was ∼3000-fold higher than the wild-type strain and similar to the addition of extracellular wild-type XIP_Sth_. These results contrast with previous results showing that transformation efficiency was much lower with natively produced XIP than with synthetic XIP added to the extracellular medium (18).

Altogether, these experiments are conclusively highlighting the mild to strong improvement of competence activation and natural transformation generated by the A5M modified peptide.

### XIP-A5M binds strongly and with a higher affinity to ComR

In order to shed light on the mechanism behind the improvement of competence activation produced by the XIP-A5M variant, different *in vitro* tests were performed to study its binding capacity to ComR_Sth_.

**Figure 3:**
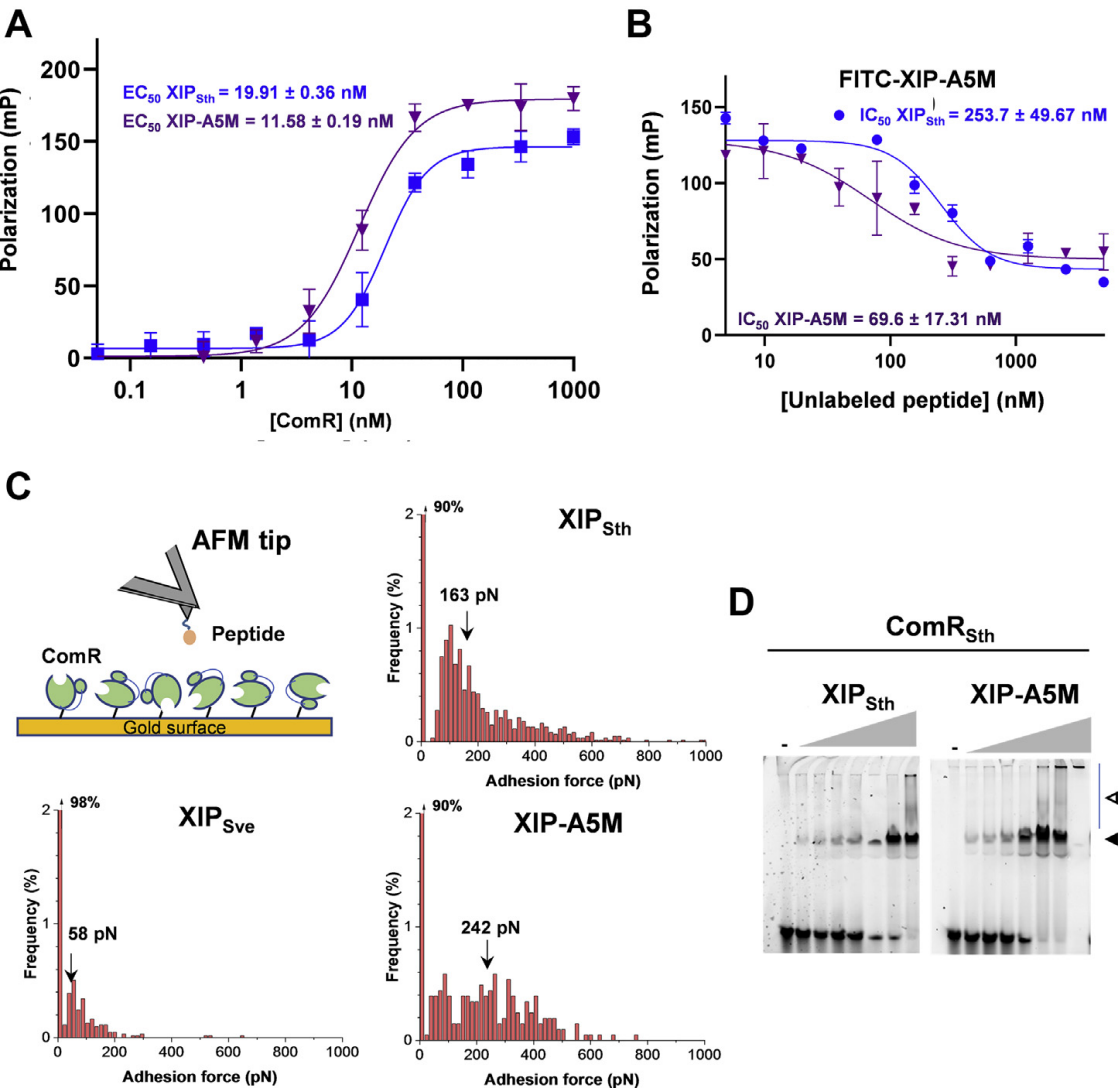
***In vitro* measurements of ComR-XIP interaction.***A*, fluorescence polarization assays. Titration was performed in the presence of a fixed concentration of FITC N-labeled XIP_Sth_ or FITC N-labeled XIP-A5M peptide (30 nM; *blue* or *purple*, respectively) and serial dilutions of ComR starting from 1 μM. Experimental values represent the mean ± SD of at least three independent replicates. Curves were fitted with the Hill equation to calculate the EC_50_ values. *B*, competition assay. Measure of decrease of fluorescent polarization signal upon addition of increasing concentrations of the unlabeled peptides XIP_Sth_ (*blue*) or XIP-A5M (*purple*) (from 0 to 5 μM) in the presence of a fixed concentration of FITC N-labeled XIP-A5M peptide (30 nM) and of ComR_Sth_ (250 nM). Experimental values represent the mean ± SD of two independent replicates. Curves were fitted to calculate IC_50_ values. *C*, atomic force spectroscopy assay. Schematic representation of AFM setup for single molecule force spectroscopy experiments where ComR_Sth_ is attached to the gold-coated glass coverslip and the XIP peptide to the cantilever. Binding force histogram of the interaction between ComR_Sth_ and XIP_Sth_ WT, XIP_Sve_, or XIP-A5M peptides. The median value (pN) is indicated in each histogram. Around 1000 curves were acquired per surface assayed from three biological replicates of each experiment where three surfaces were analyzed. *D*, electrophoretic mobility shift assay. Labeled P_*comS*_ 40-bp DNA fragments (20 ng) were incubated with a fixed concentration of ComR_Sth_ WT (1 μM) in the absence of peptide (−) or in the presence of increasing concentrations of peptide XIP_Sth_ or the variant XIP-A5M (from 0 to 2 μM).

As a first approach, its direct interaction with ComR was analyzed by fluorescent polarization (FP) assays as previously described (30, 32). For this purpose, a fixed amount of fluorophore-conjugated XIP_Sth_ WT or XIP-A5M was titrated with increasing concentrations of purified ComR_Sth_. Anisotropy measurements revealed that the EC_50_ value for XIP-A5M was twice lower than XIP_Sth_, demonstrating a higher affinity for the peptide variant (Fig. 3*A*). Moreover, competition assays were performed to test the ability of unlabeled ligands (XIP_Sth_ or XIP-A5M) to compete with the labeled form of FITC-XIP-A5M for ComR binding. The results showed a threefold lower IC_50_ value for unlabeled XIP-A5M than for the wild-type peptide, confirming the higher affinity of the modified peptide (Fig. 3*B*). As a second approach, the interaction between ComR and different peptides was probed by single molecule force spectroscopy using atomic force microscopy (AFM) (Fig. 3*C*). For this technique, the different peptides containing a 7-aa flexible linker (poly-Gly) at their N-terminus were grafted on the AFM tip, while ComR_Sth_ was attached on the surface of a gold-coated coverslip. Unlike the noncognate XIP_Sve_ that showed low binding frequency (∼2%) and weak interaction force (range from 20 to 200 pN; median value of 58 pN), XIP_Sth_ showed a broad distribution of binding forces ranging from 60 to 1000 pN with a median value of 163 pN and a binding probability of ∼10% (Fig. 3*C*). After injection of soluble XIP_Sth_, we observed a significant drop of the binding frequency to 3% (Fig. S2) that demonstrates the specificity of the ComR_Sth_-XIP_Sth_ interaction. The XIP-A5M peptide produced a binding probability of 10% comparable to the wild-type XIP_Sth_. As observed for XIP_Sth_, XIP-A5M also exhibited a broad distribution of the binding force (ranging from 60 to 780 pN); however, XIP-A5M shifted the frequency distribution from low to high binding force (median value of 242 pN) (Fig. 3*C*), suggesting a stronger interaction with ComR. In line with the above results, electrophoretic mobility shift assays showed that XIP-A5M improved the binding of ComR_Sth_ to P_*comS*_, complexing the total amount of probe at lower peptide concentrations compared with XIP_Sth_ (Fig. 3*D*).

Thus, the A5M substitution improves XIP interaction in the ComR binding pocket, resulting in a more efficient formation of the ComR·XIP complex.

### XIP-1/5 interacting residues F171–F174 are synergistically activating ComR

**Figure 4:**
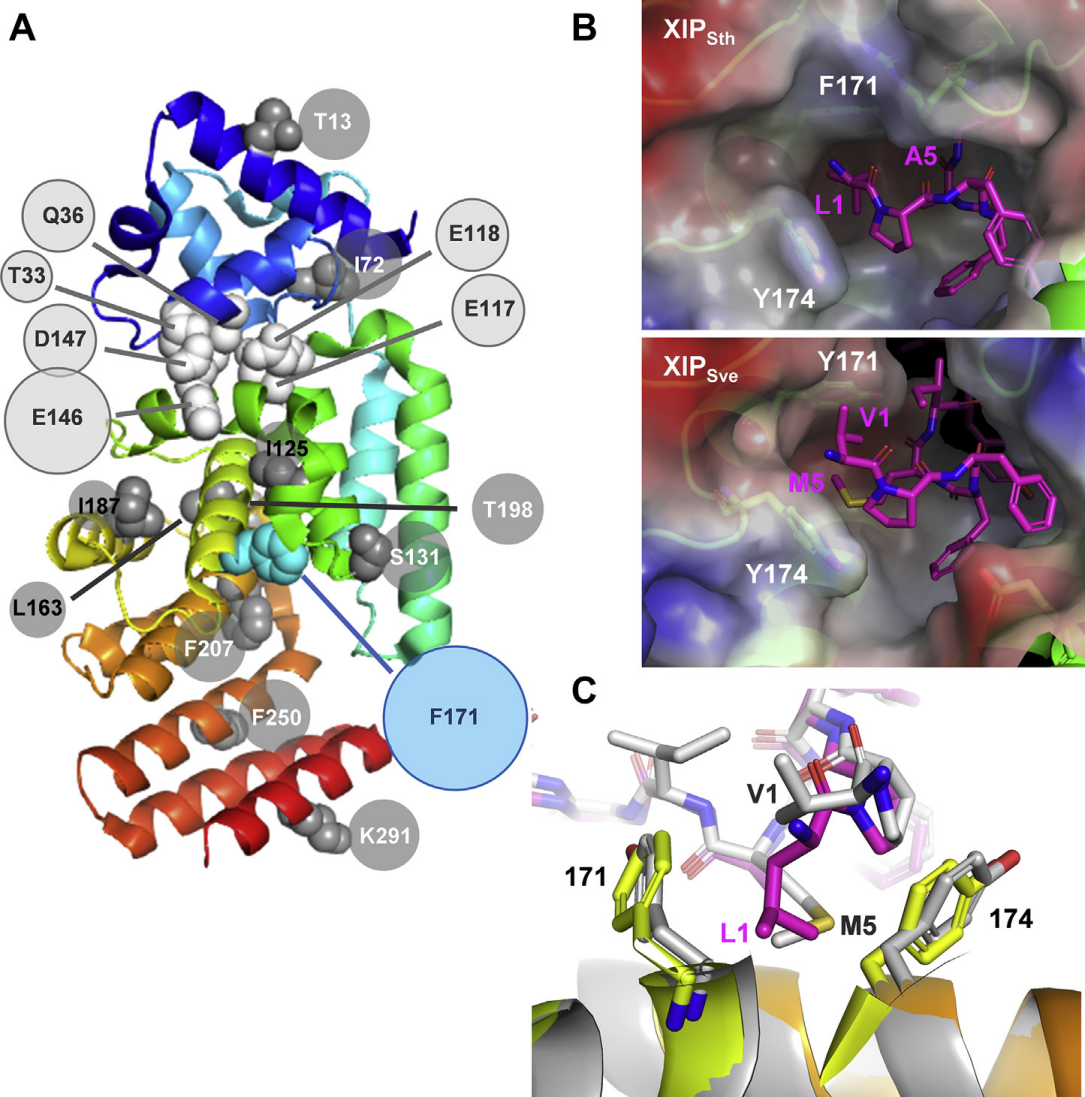
**ComR**_**Sth**_**random mutagenesis.***A*, mapping of constitutive mutations on the monomeric apo-ComR. ComR is shown as *cartoon* colored by spectrum from α1 from the HTH domain (*cyan*) to the CAP helix α16 (*red*). Residues substituted in at least two different mutants are labeled. The F171 residue, residues involved in HTH sequestration, and others along the protein are highlighted as *blue*, *white*, and *dark gray spheres*, respectively. The size of the *circle* indicates the occurrence of mutations in each position. *B*, detailed view of the hydrophobic pocket formed by ComR-171 to 174 in *S. thermophilus* (*upper panel*; PDB ID 5JUB (35)) or *S. vestibularis* (*lower panel*; PDB ID 6HUA (37)) occupied by their cognate peptide. The proteins from the ComR·XIP complexes are represented as *cartoon* and *sticks* and colored in *green*, while the peptides are in *purple*. The closed peptide-bound pockets of ComR are shown as surface colored by electrostatic potential (basic regions in *blue*, acidic region in *red*, and nonpolar residues in *white*). Residues XIP-1/5 and ComR-171/174 are labeled. *C*, detailed view of the XIP-1/5 and ComR-171/174 interaction. The 3D structures of complexes ComR_Sve_·XIP_Sve_ (colored as in panel *A*) and ComR_Sve_·XIP_Sve_ (*gray*) are superimposed. ComRs are represented as *cartoon* and XIPs in *sticks*.

In parallel to XIP mutagenesis, we performed a random mutagenesis of ComR_Sth_ using a similar screening strategy in a ComS-deficient strain with the aim to obtain mutants being natively constitutive (Fig. S3*A*). Those mutants deserve a special interest since the mutated position(s) could correspond to critical control point(s) in the ComR activating mechanism such as reported before for the release of the HTH-domain (29). We identified ∼30 mutants that showed an increased level of constitutive activation that was enhanced by the extracellular addition of the cognate peptide XIP_Sth_ (Fig. S3, *B* and *C*). Besides ∼40% of mutants that contained point mutations in residues involved in HTH-domain sequestration (*e.g.*, E118, E146, D147 or neighboring residues) (Figs. 4*A* and S3*B*), ∼60% of them displayed the substitution F171L in ComR α10 helix (alone or in combination with additional substitutions). These results strongly suggest that residue F171 is pivotal for the activating conformational change of the ComR TPR domain, which corroborates previous observations of F171-Y174 interactions with XIP-1/5 in the activation mechanism (29). In *S. thermophilus*, ComR_Sth_-F171 and Y174 form a hydrophobic cavity at the entry of the XIP-binding pocket where these two residues interact with XIP_Sth_-L1 as a linear triad of hydrophobic contacts. In *S. vestibularis*, this hydrophobic pocket (ComR_Sve_-Y171 and Y174) is occupied by XIP_Sve_-M5 with a shift of the side chain of XIP_Sve_-V1, resulting in a tetra-residue configuration (Fig. 4, *B* and *C*). Although the molecular details by which those residues influence activation are unclear, we previously showed that the double substitution F171A-Y174A in ComR_Sth_ severely reduced activation without a major impact on the binding affinity for XIP_Sth_ (29).

**Figure 5:**
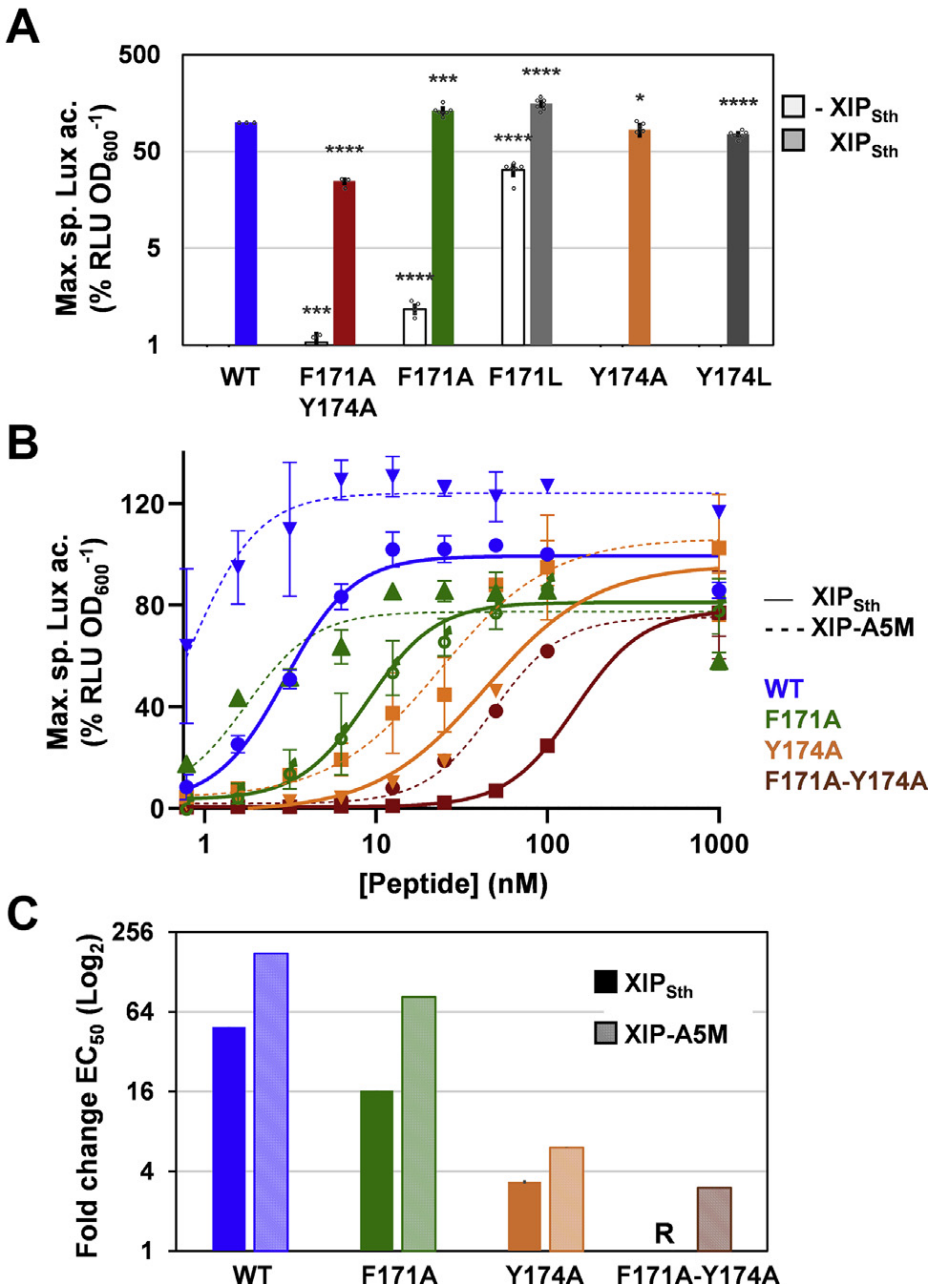
**ComR-F171 and Y174 mutants.***A*, maximum specific luciferase activity (% RLU × OD_600_^−1^) of *S. thermophilus* Δ*comS* reporter strains producing ComR_Sth_ WT (*blue*), the double mutant ComR-F171A-Y174A (*red*) or the single mutants ComR-F171A (*green*), ComR-F171L (*gray*), ComR-Y174A (*orange*), or ComR-Y174L (*dark gray*) in the absence (−XIP) or presence (+XIP) of XIP_Sth_ WT (100 nM). *B*, dose response of P_*comS*_ activity upon XIP_Sth_ WT (*solid lines*) or XIP-A5M (*dotted lines*) addition to reporter strains carrying ComR_Sth_ WT (*blue*), ComR-F171A-Y174A (*red*), ComR-F171A (*green*), or ComR-Y174A (*orange*). The monitoring and normalization of luciferase activity were performed as in Figure 2. *C*, fold changes in EC_50_ calculated from the dose response curves shown in panel *B*. The EC_50_ of the double mutant ComR-F171A-Y174A for XIP_Sth_ is used as reference (R) and normalized to 1. Decrease in fold change is representative of an increase in EC_50_. In *A*, *B*, and *C*, experimental values represent the mean ± SD of at least three independent replicates. In *A*, significant differences (ComR_Sth_ WT as reference in the presence or absence of XIP) calculated by Student unpaired *t* test are indicated (∗∗∗∗*p*-value < 0.0001, ∗∗∗*p*-value < 0.001; ∗*p*-value < 0.05).

To dissect the respective role of residues F171 and Y174 in ComR_Sth_ activation mechanism, alanine or leucine substitution of each separate residue was generated and compared with the double alanine mutant. Intriguingly, when a relatively high concentration of XIP_Sth_ was added to the medium, none of the single mutants displayed a decrease in light emission as observed for the double mutant (Fig. 5*A*). However, while ComR-Y174A and Y174L maintained a wild-type profile, ComR-F171A and F171L showed an increase in basal activation of ∼5- and ∼160-fold compared with wild-type ComR, respectively (Fig. 5*A*). For ComR-F171L, this high basal activation may explain why the signal is doubled compared with wild-type ComR when XIP_Sth_ was added. These results confirm the screening data by showing the key role played by residue 171 in the conformational change leading to spontaneous activation of ComR.

XIP_Sth_ titration of strains carrying ComR-F171A, ComR-Y174A and the double ComR-F171A-Y174A mutant highlighted that they are differently reduced in their capacity of transcriptional activation compared with wild-type ComR (∼3-, ∼15-, and ∼50-fold higher EC_50_, respectively) (Fig. 5, *B* and *C*), showing a negative synergic effect of those two mutations on ComR activation. In order to disclose the interplay between these two ComR residues and XIP, the abovementioned ComR mutants were also tested in the presence of synthetic XIP-A5M. For all the ComR mutants, XIP-A5M enhanced the luminescence signal at a lower concentration than native XIP (Fig. 5, *B* and *C*), indicating that this XIP variant does not strictly require the presence of F171 and/or Y174. However, in contrast to wild-type ComR, none of the mutants could be activated by XIP-A5M at a higher level than the native peptide, which indicates that both F171 and Y174 are required for the optimal activation of the ComR·XIP complex (Fig. 5, *B* and *C*).

These results highlight the contribution of ComR-171–174 and XIP-5 to the activation mechanism and demonstrate that those positions in ComR and XIP can embrace modifications that enhance the activation of the regulatory system.

### XIP-A5M activity is modulated by XIP-L1 and ComR-F171

The side chains of residues at positions XIP-1 and 5 display different configurations in *S. thermophilus* and *S. vestibularis*. Both XIP_Sve_-V1 and XIP_Sve_-M5 are potentially performing interactions with ComR_Sve_-Y171 and Y174 while only XIP_Sth_-L1 is interacting with ComR_Sth_-F171 and Y174 (Fig. 4, *B* and *C*).

**Figure 6:**
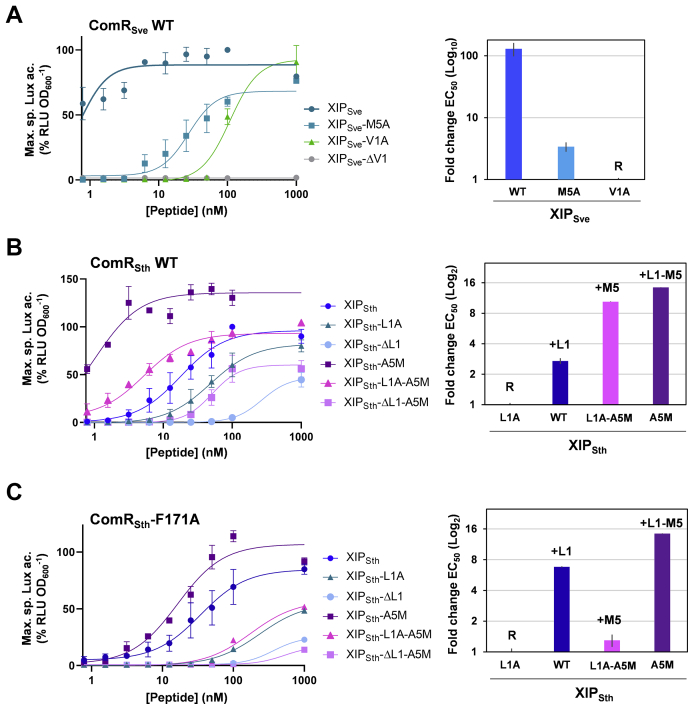
**XIP-5 and XIP-1 mutants.***A*–*C*, dose response of P_*comS*_ activity (*left panels*) or fold change in EC_50_ (*right panels*) upon addition of synthetic peptide variants carrying XIP-1 and/or XIP-5 substitutions to *S. thermophilus* reporter strains producing ComR_Sve_ WT (*A*), ComR_Sth_ WT (*B*), or ComR-F171A mutant (*C*). The monitoring and normalization of luciferase activity for dose–response curves were performed as in Figure 2. The EC_50_ condition used as reference (fixed to 1) is indicated by R. Experimental values represent the mean ± SD of at least three independent replicates.

First, we evaluated the contribution of XIP_Sve_-V1 and XIP_Sve_-M5 to the activation of ComR_Sve_. For this purpose, XIP_Sve_ variants ΔV1, V1A, and M5A were tested with a *S. thermophilus* reporter strain carrying a substitution of ComR_Sth_ by ComR_Sve_ (30). Activation was completely abolished with XIP_Sve_-ΔV1 (7 aa) and strongly decreased with XIP_Sve_-V1A (∼130-fold) and XIP_Sve_-M5A (∼40-fold) compared with wild-type XIP_Sve_ (Fig. 6*A*). This demonstrates that these two XIP positions are critical to reach the proper activation of the ComR·XIP complex in *S. vestibularis*.

Second, we hypothesized that the XIP_Sth_-A5M may adopt a similar conformation inside the peptide-binding pocket of ComR_Sth_ than observed in ComR_Sve_, thereby reducing the contribution of XIP_Sth_-L1 to the activation of the system. To test this hypothesis, several peptide variants carrying modifications in the N-terminal residue (ΔL1 and L1A) of XIP_Sth_ WT and XIP_Sth_-A5M were compared. All XIP_Sth_-ΔL1 and XIP_Sth_-L1A variants produced a decrease in light emission. The most drastic decrease was observed for XIP_Sth_-ΔL1 (7 aa, ∼90-fold less active), which suggests that the presence of a residue at position 1 is essential for the proper conformation of the peptide and its activity. However, this reduced activation was much less severe when the L1 mutation was combined with A5M (Fig. 6*B*). Using the less active 8-aa variant XIP-L1A (A1–A5) as reference, the addition of substitutions A1L, A5M, and A1L-A5M showed an increased activation of 2.7, 10.4, and 14.4-fold, respectively (Fig. 6*B*). This shows that both leucine L1 and methionine M5 contribute additively to a better ComR_Sth_ activation but with a more predominant role for M5.

Finally, we evaluated the whole range of XIP_Sth_ variants with the ComR-F171A mutant. Notably, when A5M substitution was combined with L1A or ΔL1 mutations, the stimulating effect of A5M was lost in the F171A mutant compared with wild-type ComR_Sth_ (Fig. 6*C*). When using the XIP-L1A as reference, the addition of substitutions A1L, A5M, and A1L-A5M showed an increased activation of 6.8, 1.3, and 14.4-fold, respectively (Fig. 6*C*). While L1 and M5 displayed an additive effect in the activation of wild-type ComR_Sth_, those residues are acting synergistically to amplify the activation of the F171A mutant. Moreover, an interaction between XIP_Sth_-M5 and F171 seems required to observe the boosting effect of M5 when L1 is mutated.

Altogether, these data show that XIP_Sth_-L1 and XIP_Sth_-M5 are collaborating in the activation mechanism of ComR_Sth_, most probably by interacting with ComR_Sth_-171 to 174 residues as observed in the crystal structure of ComR_Sve_.

### ComR-171/174 – XIP-1/5 interactions remodel a network of aromatic–aromatic interactions

**Figure 7:**
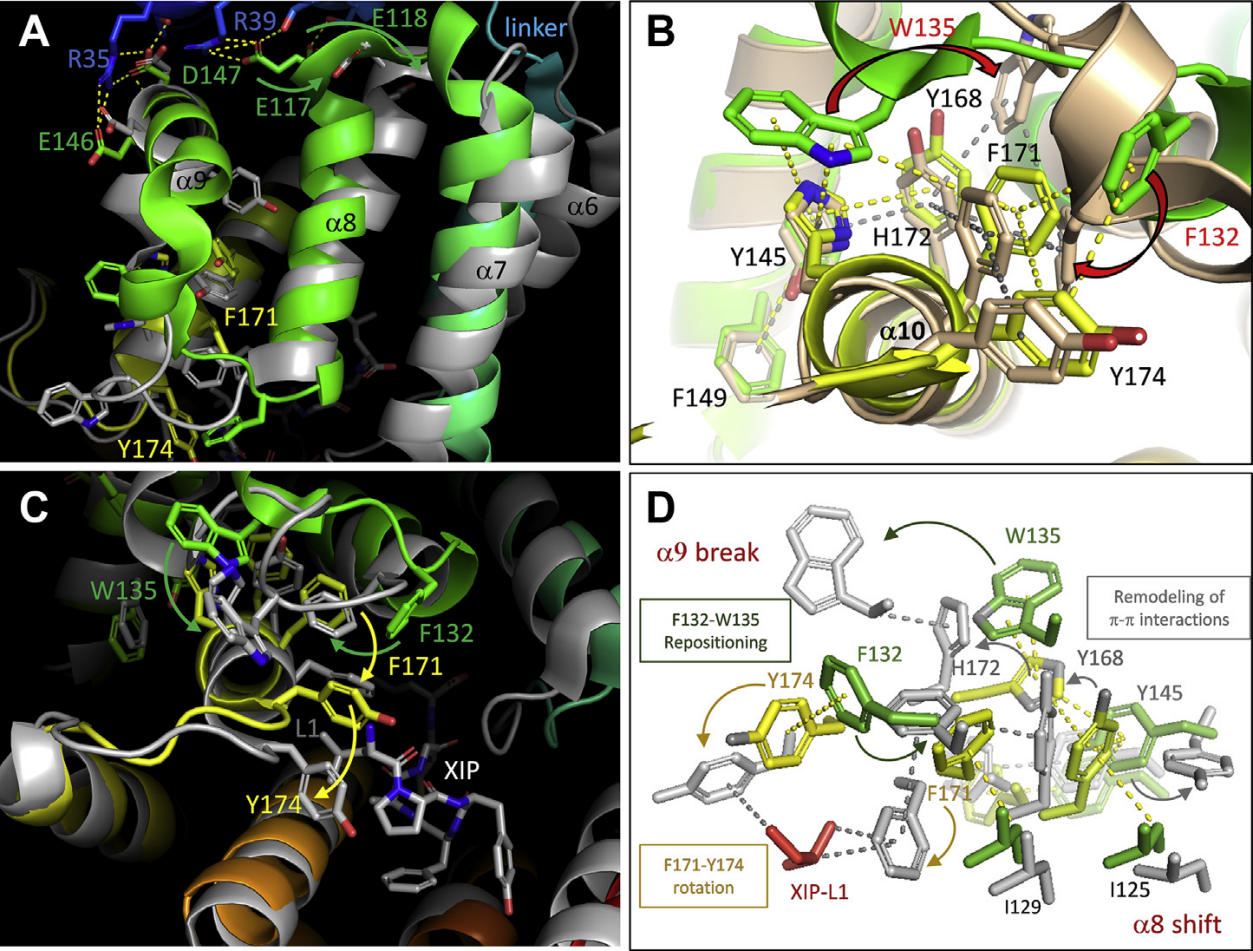
**Remodeling of a network of aromatic–aromatic interactions in ComR activation.***A*, XIP-driven reorganization of TPR-1 (α6–α7) and TPR-2 (α8–α9). Apo-ComR_Sth_ (colored by spectrum; PDB ID 5JUF (33)) and ComR_Sth_·XIP_Sth_ complex (*gray*; PDB ID 5JUB (35)) are superimposed. At the *top*, the helix-α8 reorientation is responsible of the break of R39-R51/E117-E118 salt bridges, which contributes to helix−α9 destabilization needed for the HTH domain release (break of R35/E146-D147 salt bridges). At the *bottom*, the remodeling of loop (α8–α9) and the reorientation of aromatic residues F171-Y174 are observed. *B*, F171-Y174 aromatic–aromatic interactions in apo-ComR. The major form (colored by spectrum; PDB ID 5JUF (33)) and a minor form (*beige*; Chain A, PDB ID 6QER (34)) of apo-ComR_Sth_ are superimposed. The network of observed aromatic–aromatic interactions is modified by the repositioning of F132 and W135 from loop α8 to α9 (*red arrows*). Key residues from helix α9 (Y145 and F149) and helix α10 (Y168, F171, H172 and Y174) are indicated. *C*, XIP-driven reorganization of F171-Y174 aromatic–aromatic interactions. Apo-ComR_Sth_ (colored by spectrum; PDB ID 5JUF (33)) and ComR_Sth_·XIP_Sth_ complex (*gray*; PDB ID 5JUB (35)) are superimposed. The rotation of the lateral chains of F171-Y174 in interaction with XIP-L1 and the repositioning of F132 and W135 from loop α8 to α9 are highlighted. *D*, detailed view of XIP-driven reorganization of aromatic lateral chains surrounding F171-Y174. Apo-ComR_Sth_ (colored by spectrum; PDB ID 5JUF (33)) and ComR_Sth_·XIP_Sth_ complex (*gray*; PDB ID 5JUB (35)) are superimposed. The reorientation/repositioning of shown residues participates to helix-α8 shift and helix-α9 break required for HTH domain release. Interactions were mapped using Arpeggio (http://biosig.unimelb.edu.au/arpeggioweb/) (52).

We previously reported that apo-ComR activation by XIP relies on the recruitment of TPR-1 (loop α6–α7) and the CAP helix, the transmission of the shift of helix α7 to an helix-α8 reorientation, which participates to destabilization of helix α9 (break of R39-R51/E117-E118 salt bridges), and finally helix-α9 disruption at position D147 that is needed for HTH-domain release (break of R35/E146-D147 salt bridges) (Fig. 7*A*) (29, 30). However, the results presented here revealed that a key part of the activation mechanism is taking place on the other side of the TPR domain through the interplay between XIP-1/5 and the duo F171-Y174 of helix α10 (Fig. 4).

Using comparative structural analyses and extensive mapping of interactions, we investigated the network of interactions involving residues F/Y171-Y174 in ComR apo-forms and ComR·XIP complexes. The crystal structure of apo-ComR_Sth_ revealed a major (PDB ID 5JUF (33)) and a minor form (Chain A, PDB ID 6QER (34)), in which loop α8 to α9 displayed a completely different conformation (Figs. 7*B* and S4). In the alternative conformation, a large network of predicted aromatic–aromatic interactions that includes residues from loop α8 to α9 (F132_Lα8–α9_ and W135_Lα8–α9_), and other residues from helix α9 (Y145_α9_ and F149_α9_) and helix α10 (Y168_α10_, F171_α10_, H172_α10_ and Y174_α10_) is completely modified, highlighting the high plasticity of loop α8 to α9 residues in Apo-ComR stabilization (Figs. 7*B* and S5). XIP binding through XIP-L1/F171-Y174 interactions induces a new conformational change in loop α8 to α9, which further remodeled this network (Figs. 7*C* and S5; PDB ID 5JUB (35)). The interaction with XIP-L1 (assisted by relay interactions with XIP-P2-F4-Y3) leads to a ∼90° rotation of lateral chains of the F171_α10_-Y174_α10_ duo and a disruption of aromatic–aromatic interaction(s) with F132_Lα8–α9_. This allows the repositioning of F132_Lα8–α9_ at the place of F171_α10_ to form a novel cluster of predicted π-π interactions, including Y168_α10_ and F171_α10_ (Figs. 7*D* and S5). Concomitantly, the interaction Y168_α10_-I125_α8_ is weakened and W135_Lα8–α9_-H172_α10_-E142_α9_/Y145_α9_-Y168_α10_ interactions are disrupted. Those two events participate in helix-α8 shift and destabilization of helix α9, respectively, both required for HTH-domain release (Figs. 7, *C* and *D* and S5). A similar effect could be predicted for the XIP_Sve_-V1-M5 duo interacting with ComR_Sve_ Y171-Y174 as all the key residues cited above and the predicted network of aromatic–aromatic interactions are largely conserved in ComR of *S. vestibularis* (PDB ID 6HU8 (36) and 6HUA (37)) (Fig. S5). However, in this case and which could be hypothesized for XIP_Sth_-A5M, the interaction of XIP-1 with F/Y171-Y174 is reinforced by a predicted sulfur–π interaction provided by the methionine at residue XIP-M5 (Fig. S5).

Altogether, these structural comparisons highlight the critical importance of the local remodeling of the network of aromatic–aromatic interactions at the entry of the peptide-binding pocket to allow proper ComR activation.

## Discussion

Unveiling the detailed activation mechanism of ComRS systems is an important step toward a better control of DNA transformation as well as understanding their divergent evolution in the context of cell-to-cell communication in streptococci. This work significantly contributes to both aspects, highlighting a critical trigger point involving interactions between the XIP pheromone and its sensor in the activation mechanism.

**Figure 8:**
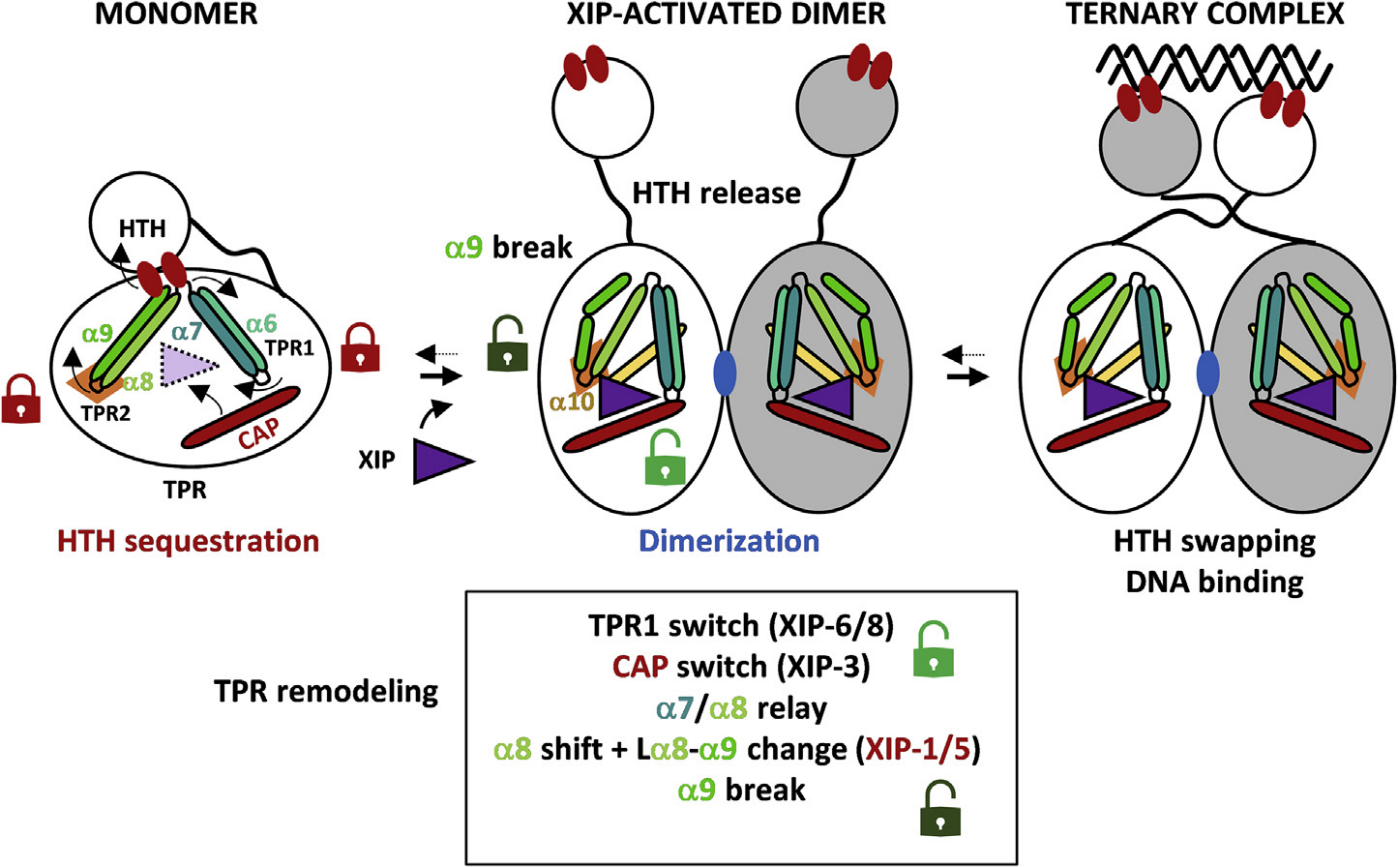
**Refined model of ComR activation mechanism.** The HTH and TPR domains of the protein are shown with the flexible linker. TPR-1 (α6–α7), TPR-2 (α8–α9), α10, and CAP helix, directly implicated in XIP binding and release of the ComR locked state, are shown as *sticks* and labeled. Helices are colored as in Figure 4. *Red* and *blue ellipses* represent residues involved in HTH sequestration and TPR dimerization, respectively. The *orange diamond* represents local aromatic–aromatic interactions that are remodeled through XIP-1/5-F/Y171-Y178 interactions.

By analogy, the pheromone–sensor interaction is often simplified and viewed as a selective key–pinhole interaction. However, the situation appears more complex as shown here for the XIP-ComR interaction where XIP is a key with multiple tines whose contacts with its keyhole in ComR will unlock multiple doors of differential importance to allow its dimerization and release of DNA-binding domains. We propose a refined model of the ComRS activation mechanism where the TPR domain is double-locked for its conformational change (Fig. 8, Movies S1 and S2). We previously revealed one of the two unlocking steps involving the toggle switch of TPR-1 (XIP-6 and XIP-8 interactions), CAP recruitment (XIP-3 interaction), and a relay toward a helix-α8 shift contributing to the HTH-domain release (29, 30). Here, we shed light on a second unlocking step that involves the reorganization of TPR-2 (helix-α8 shift and helix-α9 break) through a local remodeling of a network of secondary interactions resulting from contacts with XIP-1/5. While the separate blocking of each remodeling step by targeted mutations of involved residues (*i.e.*, K100A and F171A-Y174A, respectively) was previously shown to strongly decrease XIP-mediated activation (29), the second conformational change is of major importance as a single residue mutation (*i.e.*, F171L) nearly bypassed XIP requirement for activation (Fig. 5*A*). Moreover, the level of constitutive activation is higher than just releasing sequestration of the HTH domain as reported before (29), suggesting a preferential stabilization of the TPR-domain in its active conformation that could for instance promote its dimerization.

Our ComR_Sth_ mutagenesis of aromatic residues F171 and/or Y174 involved in this second unlocking step showed that the progressive decrease of hydrophobic contacts with XIP-1/5 negatively affects XIP activation capacity in a synergistic manner (F171A-Y174A << Y174A < F171A < ComR_WT_) (Fig. 5*C*). Reciprocally, the presence of alanine residues at XIP position 1 and/or 5 in XIP_Sth_ or XIP_Sve_ generated a similar negative effect (Fig. 6*B*), confirming their complementary interdependence in the remodeling of the network of secondary interactions. This predicted network corresponds to a very large cluster of π-π (12–14) and C-H···π (9–11) interactions, buried in the hydrophobic core of ComR and interconnecting helices α8, α9, and α10 (Fig. S5). This network is remodeled upon XIP interaction, which allows the rearrangement of loop α8 to α9 and TPR conformational change (Fig. S5). A range of mutagenesis works have shown that these aromatic clusters are very sensitive to destabilization by simple conservative mutation (*e.g.*, aromatic residue to leucine) or subtle reorientation of aromatic rings (38, 39, 40, 41). Similarly, our random mutagenesis revealed that the ComR-F171_α10_ residue is pivotal for maintaining the nonactive status of the apo form. Its substitution by a conservative leucine residue has a dramatic effect on the differential stability between the TPR-domain conformations, leading to a constitutively active ComR. Moreover, since the F171A mutant displayed a lower constitutive activation, this active conformation is probably preferentially stabilized by hydrophobic contacts(s) involving L171. A possible scenario is the positioning of L171 in a similar configuration than F171 in the activated form with its stabilization through interactions with F132_Lα8–α9_ and Y168_α10_, which is probably less efficient with an alanine residue (Fig. S5). In this case, the L171 mutation may somehow mimic the XIP-L1/ComR-F171 interaction, resulting in a similar reorganization of the aromatic–aromatic interaction network as reported for the active conformation (Fig. S5). Our mutagenesis work also showed that Y174 seems to play a less important role in the differential stabilization between apo and holo states, probably due to the external position of this residue in the network (Figs. 5, *A* and *B* and S5). It is interesting to note that mutating residue I125_α8_ into leucine, which interacts with Y168_α10_ and thus participates in the network by anchoring of α8 to α10 (Fig. 7*D*), also leads to a constitutive activation but at a lower level than F171L (Figs. S3 and S5). In contrast to the negative effect on protein functionality generally assigned to disruption of aromatic clusters (38, 39, 41), our results show that the fine-tuning of hydrophobic contacts between XIP and ComR-F171-Y174 in the peptide-binding pocket positively remodel ComR for its activation.

The XIP semirational mutagenesis used during this work also revealed that the efficiency of ComRS activation can be significantly improved by a modified pheromone with a reactivity in the sub-nanomolar range and a dramatic increase in DNA transformation efficiency (Fig. 2, *A* and *D*). To our knowledge, such improvement of the activation of a member of the (R)RNPP family has never been reported so far. The higher activation of ComR_Sth_ by the presence of a single substitution of an alanine by a methionine at XIP-5 results probably from mimetic interactions, which are taking place at the entry of the XIP-binding pocket in *S. vestibularis* ComR (Fig. 4, *A* and *B*). Since methionine is more hydrophobic than alanine, XIP-A5M is less stable in water and its desolvation upon binding to ComR may account for its increased affinity. Indeed, a difference in desolvation energy between 1.2 and 1.4 kcal/mol is expected upon alanine to methionine substitution (42), resulting in a maximal seven- to ten-fold increase in affinity. The flexibility of the linear methionine side chain should help accommodating the residue in the binding pocket and minimizing steric hindrance in the complex. Besides flexibility and hydrophobicity, methionine is also quite unique in its ability to perform sulfur–π interaction(s) that may bring additional stabilization of ∼1.0 to 1.5 kcal/mol (43). Such interaction is predicted between XIP_Sve_-M5 and ComR_Sve_-Y174 (Fig. S5) and may take place similarly in ComR_Sth_ although it may require structural rearrangement. Sulfur–π interactions have been reported to strongly contribute to protein stabilization, receptor–ligand or protein–protein interactions, and more recently to ion channel gating (43, 44, 45). Finally, the triad aromatic-Met-aromatic (named Aro-Met-Aro) has recently been identified as a novel motif in numerous crystal structures (46). Here, an intermolecular Aro-Met-Aro bridging interaction may further stabilize the XIP-M5 and ComR-Y/F171-Y174 complex.

ComRS systems have divergently evolved in streptococci with the apparition of pherotypes that can largely differ, even between members of the same *streptococcus* group (19). In salivarius streptococci, two well-separated pherotypes are found between *S. thermophilus*/*S. salivarius* (named type Ia) and *S. vestibularis* (type Ib) (prototypes in Fig. 1*B*) (19, 30). Although no XIP type Ia with a methionine at position 5 has been found until now, some XIPs exhibit a threonine at that position in type Ia or an isoleucine in type Ib (30). Interestingly, the natural variation A5T is neutral compared with the most represented A5 residue in XIP type Ia (Fig. 2*C*). However, in a similar situation to XIP_Sth_-A5M, the A5I substitution improved transcriptional activation but at a lower level than A5M (Fig. 2*C*). These observations corroborate the relative importance of XIP-1/5 in the activation of ComR_Sve_ *versus* ComR_Sth_ (Fig. 6, *A* and *B*) and suggest that pheromones or ComR-pheromone couples found in nature are not necessarily the most active ones. In the same vein, we observed a growth defect when the more active peptide is added to the medium or is natively produced (Fig. 2*B*). It is important to recall that competence development is a tightly controlled process that is generally activated during a short time window (13). It is also well documented that the process is not only energetically expensive but can also disturb cell division and chromosome integrity when dysregulated (9, 13). These physiological disturbances probably explain evolutionary constrains on the ComRS system. They prevent hyperactive systems in order to minimize the decrease in fitness when competence is over-triggered and ensure its tight control.

To conclude, our results shed light on a novel trigger point in the ComR activation mechanism and its evolutionary consequences for the fine-tuning of competence control. Besides, this work also offers biotechnological opportunities to better stimulate natural transformation for engineering food-associated streptococci or improving peptide-based expression systems. As ComR stimulates the production of antimicrobials (bacteriocins) in many streptococci (31, 32), optimized pheromone might alternatively be exploited to bolster interspecies predation and kill pathogenic bacteria at the infection site.

## Experimental procedures

### Bacterial strains, plasmids, and oligonucleotides

Bacterial strains, plasmids, and oligonucleotides used in this study are listed in Tables S1 and S2. The primers used in this study were purchased from Eurogentec.

### Growth conditions

*S. thermophilus* LMD-9 and derivatives were grown at 37 °C without shaking in M17 broth (Difco Laboratories Inc) or in CDM (47) supplemented with 1% glucose [w/v] (M17 and CDMG broth, respectively). *Escherichia coli* was grown in LB medium with shaking at 37 °C. When required, chloramphenicol (3.5 or 5 μg ml^−1^ for *S. thermophilus*), erythromycin (2.5 μg ml^−1^ for *S. thermophilus*), or ampicillin (200 μg ml^−1^ for *E. coli*) was added to the media. Plates inoculated with *S. thermophilus* cells were incubated anaerobically (BBL GasPak systems, Becton Dickinson) at 37 °C.

### Preparation of XIP peptides

Synthetic XIP octapeptides, polyG-XIP octapeptides, and FITC N-labeled nonapeptides were supplied by Peptide 2.0 or GeneScript (Table S3). They were resuspended in bi-distillated water, except FITC N-labeled nonapeptides that were solubilized in 100% dimethyl sulfoxide (DMSO) (vol/vol). Final concentration was quantified using a Nanodrop apparatus (Thermo Fisher Scientific).

### Natural DNA transformation

To induce competence, overnight *S. thermophilus* precultures grown in CDMG were diluted in semiskimmed milk at a final OD_600_ of 0.05. After an incubation of 75 min at 37 °C, 1 μM of XIP_Sth_ WT and linear DNA fragments were added. Cells were grown for 4 h at 37 °C before plating on selective M17G agar and incubation in anaerobic conditions. Positive candidates were confirmed by streaking on selective plates and verified by PCR and sequencing (18, 48).

Transformation efficiency assays were performed as reported above by adding 1 μg of multimeric and circular plasmid pGhost9-core, together with XIP_Sth_ (100 nM) when needed. All incubation steps were performed at 30 °C to allow plasmid replication. The plasmid was purified from *E. coli* using a Maxiprep Kit for low-copy plasmids (Thermo Fisher Scientific) following manufacturer’ instructions. Transformation efficiency corresponds to the total number of transformants (erythromycin-resistant colony-forming units) per ml.

### Construction of comS and comR mutant strains

*S. thermophilus* strain LL30 (LMD-9 derivative) expressing *comS-11aa* was constructed as follows. Four PCR fragments with the following features were joined to replace *comS* by *comS-11aa*: fragment 1 with *comR* and P_*comS*_ (primers #1 and #2), fragment 2 with the *comS*_Δ*2–13*_ (primers #3 and #4), fragment 3 with the P_*32*_*-cat* cassette (primers #6 and #7), and fragment 4 with the ∼1-kb *comS* downstream region (primers #5 and #25). With the exception of the P_*32*_*-cat* cassette amplified from strain LF134 (19), strain LMD-9 was used as template to amplify the other fragments.

Strain LL31 containing a replacement of *comS* by a P_*comS*_-*cat* fusion was constructed by joining two PCR fragments: fragment 1 with *comR*-P_*comS*_ (primers #1 and #12; LMD-9 as template) and fragment 2 with the promoter-less *cat* gene (primers #11 and #25; LF134 as template).

Strains LL32 and LL33 expressing *comS-24aa-A5M* and *comS-24aa-L1V-A5M*, respectively, were constructed by joining two PCR fragments: fragment 1 with *comR*-P_*comS*_ and *comS′* encoding N-terminal ComS-16aa (primers #1 and #8; LMD-9 as template) and fragment 2 with point mutation(s) A5M without or with L1V (primers #9 or #10, and #25, respectively; LL30 as template).

Strains LL40, LL42, LL42, and LL43 expressing *comR-F171A*, *comR-F171L*, *comR-Y174A*, and *comR-Y174L*, respectively, were constructed by joining two PCR fragments: fragment 1 with a ∼1-kb *comR* upstream region and the 5′end of *comR* (primers #24 and #17, #19, #21, or #23, respectively; LMD-9 as template), and fragment 2 with the 3′ end of *comR*, *comS* substituted by P_*32*_*-cat*, and ∼1-kb downstream region of *comS* (primers #31 and #16, #18, #20, or #22, respectively; LF134 as template). Internal primers enclosed the point mutation in each case.

The different fragments were joined by overlapping PCR using external primers and the full-length product was transformed by natural transformation in the reporter strain LF121 (P_*comS*_*-luxAB*) for chromosomal replacement by double homologous recombination (19). For strain LL31, transformants were selected on plates containing both chloramphenicol and XIP_Sth_ 1 μM. Then, this strain was validated as chloramphenicol sensitive in absence of XIP_Sth_.

### Semirational XIP mutagenesis in *S. thermophilus*

In order to perform the peptide screening, strain LL34 containing both P_*comS*_*-cat* and P_*comS*_*-comS-11aa* was constructed. Three PCR fragments with the following features were joined: fragment 1 with *comR* and P_*comS*_*-cat* (primers #1 and #7; LL31 as template), fragment 2 with P_*comS*_*-comS-11aa* (primers #14 and #15; LL30 as template), and fragment 3 with ∼1-kb *comS* downstream region (primers #13 and #25, LMD-9 as template). Overlapping PCR and natural transformation in LF121 were performed as reported above.

To generate the DNA library encoding semirandomized peptide variants, two PCR fragments were jointed: fragment 1 with *comR*-P_*comS*_*-cat* (primers #1 and #33; LL34 as template) and fragment 2 with the semi-randomized DNA stretch and ∼1 kb *comS*-downstream region (primers #32 and #25; LL34 as template). Primer #32 included a semidegenerated sequence allowing any amino acid exchange between XIP_Sth_ and XIP_Sve_ sequences (8 aa). After overlapping PCR to join the fragments, the final PCR product was transformed in the reporter strain LF121. Chloramphenicol-resistant candidates were restreaked on selective medium and analyzed by sequencing and luciferase activation.

### Random *ComR* mutagenesis in *S. thermophilus*

To generate the *comR* library, random ComR_Sth_ mutagenesis was achieved by error-prone PCR allowing 0 to 3 mutations per kb (49). The ComR mutant library was produced by amplifying the *comR*_*Sth*_ gene with primers #28 and #29 (LMD-9 as template). The library was included in a final overlapping PCR product obtained from three PCR fragments: fragment 1 with a ∼1-kb *comR* upstream region (primers #24 and #30, LMD-9 as template), fragment 2 with the *comR*_*Sth*_ random library, and fragment 3 with a ∼1-kb downstream region of P_*comS*_*-cat* cassette (primers #31 and #25; LL31 as template). The final PCR product carrying *comR* mutants was transformed in the reporter strain LF121 (19). Natural transformation was performed as reported above and transformants were selected on chloramphenicol (3.5 μg ml^−1^), restreaked on selective medium with and without XIP_Sth_ 1 μM, and tested for luciferase activity.

### Measurements of luciferase activity

Luciferase assays were performed as previously described (19). Overnight precultures were diluted to a final OD_600_ of 0.05 and culture samples incubated in a sterile covered white microplate with a transparent bottom (Greiner). Growth and luciferase activity (expressed in relative light units [RLUs]) of the cultures were monitored after addition or in absence of synthetic XIP peptide at 10 min intervals during at least 8 h in a multiwell plate reader (Hidex Sense, Hidex). When peptides were titrated, 0, 0.78, 1.56, 3.12, 6.25, 12.5, 25, 50, 100, and 1000 nM were added to the medium. In order to calculate EC_50_ values (peptide concentration for a half maximum response), maximum luciferase activity values were fitted to Hill equation:

$$\text{L}=\frac{{\text{L}}_{\max}\times {\left[\text{XIP}\right]}^{n}}{{\left(EC_{50}\right)}^{n}+{\left[\text{XIP}\right]}^{n}}$$

where L is the luciferase activity, [XIP] is the XIP concentration, and *n* is the Hill coefficient. Curves were fitted with the GraphPad Prism software v.9.0.1 (GraphPad Software, Inc).

### ComR purification

*E. coli* strain TOP10 (Invitrogen) electrotransformation of plasmid pBADcomRSth-strep, purification of ComR_Sth_-StreptagII protein, and protein storage were performed as described previously (30). Protein purity was analyzed by SDS-PAGE and protein concentration was measured using a Nanodrop apparatus (Thermo Fisher Scientific).

### Fluorescence polarization assays

FP assays were performed as previously described (30, 32). Threefold serial dilutions of purified ComR (initial concentration of 1 μM) were mixed with a FITC N-labeled version (9 aa) of XIP peptides (ILPYFAGCL and ILPYFMGCL) at a fixed concentration of 30 nM. The samples were incubated for 10 min at 30 °C in black 96-well plates (Greiner). Anisotropic measurements were performed in a multiwell plate reader (Hidex Sense, Hidex) in polarization mode with 485/10-nm and 535/20-nm excitation and emission filters, respectively. EC_50_ values of curves that reached saturation were obtained by fitting the plots to the Hill equation:

$$\text{F}=\frac{{\text{F}}_{\max}\times {\left[\text{XIP}\right]}^{n}}{{\left(EC_{50}\right)}^{n}+{\left[\text{XIP}\right]}^{n}}$$

where F is the polarized fluorescence, [XIP] is the XIP concentration, and *n* is the Hill coefficient. Curves were fitted with the GraphPad Prism software v.9.0.1 (GraphPad Software, Inc).

In the case of competition assays, a fixed concentration of ComR (250 nM) and FITC N-labeled XIP peptide (30 nM) were incubated with twofold serial dilutions of unlabeled XIP variant (initial concentration of 5 μM). Plots were fitted by using GraphPad Prisma software to calculate IC_50_ values (half-maximal inhibitory peptide concentration).

### Single-molecule force spectroscopy

Gold-coated glass coverslips and cantilevers (OMCL-TR4, Olympus Ltd; nominal spring constant ∼0.02 N m^−1^) were immersed overnight in an ethanol solution containing 1 mM of 10% 16- mercaptododecahexanoic acid/90% 1-mercapto-1-undecanol (Sigma), rinsed with ethanol and dried with N_2_. Substrates and cantilevers were then immersed for 30 min into a solution containing 10 mg ml^−1^ *N*-hydroxysuccinimide (NHS) and 25 mg ml^−1^ 1-ethyl-3-(3-dimethylaminopropyl)-carbodiimide (EDC) (Sigma), rinsed with Ultrapure water (ELGA LabWater), incubated with 0.1 mg ml^−1^ ComR or polyG-XIP_Sth_, polyG-XIP-A5M, or polyG-XIP_Sve_ peptides for 1 h, rinsed further with PBS buffer, and then immediately used without dewetting.

For experiments of single-molecule force spectroscopy, ComR-functionalized surfaces and functionalized cantilevers with polyG-peptides were prepared as described above. Measurements were performed at room temperature in PBS with a Force Robot 300 AFM (JPK Instruments). Multiple (32 × 32) force–distance curves were recorded on areas of 500 by 500 nm^2^ with an applied force of 250 pN, a constant approach, and retraction speed of 1000 nm s^−1^. Histograms were generated by considering, for every curve, the force and the distance of the last rupture event. The spring constants of the cantilevers were measured by the thermal noise method (50). Data were analyzed with the data processing software from JPK Instruments.

### Electrophoretic mobility shift assays

EMSA assays were performed as previously described (19, 30). A fixed concentration of purified ComR protein (1 μM) was mixed with twofold serial dilutions of the XIP variant (initial concentration of 2 μM) together with a 40-bp dsDNA fragment (20 ng) carrying the ComR box of PcomS coupled to the Cy3 fluorophore. Negative controls were performed in absence of XIP. Mix was incubated at 37 °C for 10 min prior to analysis on a native 4 to 20% gradient gel (iD PAGE gel; Eurogentec) and DNA complexes were detected by fluorescence on the Ettan DIGE Imager with bandpass excitation and emission filters of 540/25 and 595/25 nm, respectively (GE Healthcare). Double-stranded DNA fragment was obtained from annealing of single-stranded Cy3-labeled (at 5′ end) and unlabeled oligonucleotides.

### Multiple sequence alignments, phylogenetic analysis, and structure visualization

Multiple alignment of screened XIP variants was generated with Clustal Omega (https://www.ebi.ac.uk/Tools/msa/clustalo/) (51). The formatting of the alignment was performed with CLC Main Workbench 7 (https://www.qiagenbioinformatics.com/). The figures with structural elements were prepared by using the graphic software PyMol (http://www.pymol.org/).

## Data availability

All data are contained within the article and the supporting information.

## Supporting information

This article contains supporting information (18, 19, 29, 30, 33, 34, 35, 36, 37, 52).

## Conflict of interest

P. H. and Y. F. D. are research directors at FNRS. All other authors declare that they have no conflicts of interest with the contents of this article.
