# Supplementary material for: Coevolution of the bacterial pheromone ComS and sensor ComR fine-tunes natural transformation in streptococci
Source: J Biol Chem. 2021 Oct 27;297(6):101346. doi: 10.1016/j.jbc.2021.101346 (PMC8605241; doi:10.1016/j.jbc.2021.101346)
Supplement: Supplemental Figures S1–S5 and Tables S1–S3 [file mmc3.docx]

**Supporting information**

**Coevolution of the bacterial pheromone ComS and sensor ComR fine-tunes natural transformation in streptococci**

Laura Ledesma-García, Imke Ensinck, Denis Dereinne, Felipe Viela, Johann Mignolet, Yves F. Dufrêne, Patrice Soumillion, Sylvie Nessler, and Pascal Hols

**Supplemental figures:**

**Fig. S1.** Controls for XIP mutagenesis

**Fig. S2.** Controls for atomic force spectroscopy assay

**Fig. S3.** ComR random mutagenesis

**Fig. S4.** Remodeling of loop α8-α9 and aromatic-aromatic interactions in apo-ComR_Sth_

**Fig. S5.** Topology of the network of aromatic-aromatic interactions in apo-ComR_Sth_, ComR_Sth_**·**XIP_Sth_ complex, model of ComR_Sth_-F171L, apo-ComR_Sve_, and ComR_Sve_**·**XIP_Sve_ complex

**Supplemental movies:**

**Movie S1.** TPR-domain remodeling of Apo-ComR_Sth_ upon XIP_Sth_ binding

**Movie S2**. TPR-domain remodeling of Apo-ComR_Sve_ upon XIP_Sve_ binding

**Supplemental tables:**

**Table S1.** Bacterial strains and plasmids used in this study

**Table S2.** Primers used in this study

**Table S3.** Synthetic peptides used in this study

**Supplemental figures**

**
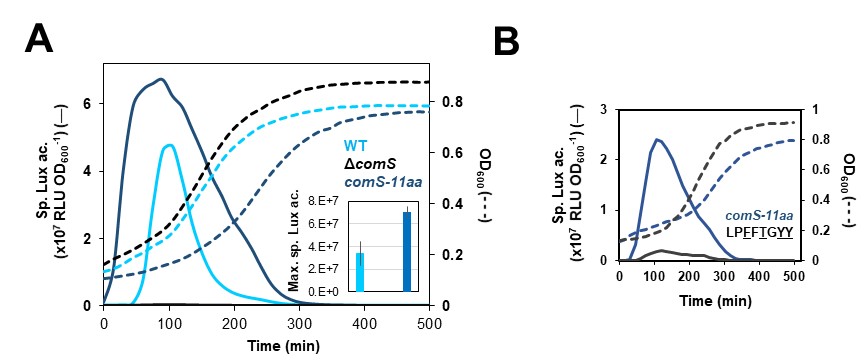
**

**Fig. S1. Controls for XIP mutagenesis.** *A*, kinetics of luciferase activation (solid lines) and growth curves (dotted lines) of reporter strains producing ComS-24aa WT (light blue), ComS-11aa WT (blue), or a Δ*comS* mutant strain (black). Inset shows the maximum specific luciferase activity (RLU OD_600_^−1^) of each strain*.* Experimental values represent the mean ± SD of at least three independent replicates. *B*, kinetics of luciferase activation (solid lines) and growth curves (dotted lines) of reporter strains producing ComS-11aa*-*LPFFTGYY (dark grey) used as negative control and ComS-11aa WT (blue).


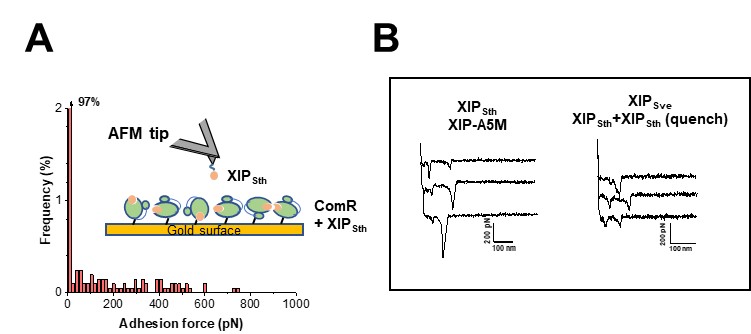


**Fig. S2. Controls for atomic force spectroscopy assay.** *A*, schematic representation of AFM set up where XIP_Sth_ is added to the solution. *B*, representative curves of ComR interaction with XIP_Sth_ or XIP-A5M (left); or ComR interaction with XIP_Sve_ or XIP_Sth_ when the same peptide is added to the solution as quencher (right).

**
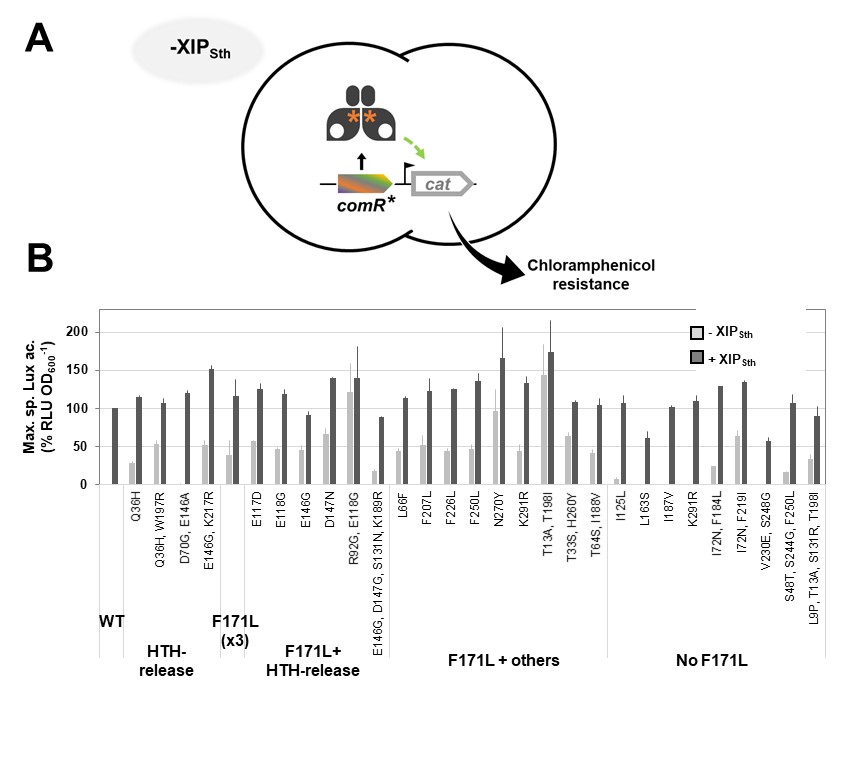
**

**Fig. S3. ComR random mutagenesis.** *A*, screening strategy. A library of random ComR mutants (error-prone PCR, rainbow arrow) under the control of the native *comR* promoter is expressed into a reporter strain (P*_comS_*-*luxAB* fusion) where the *comS* ORF has been substituted by a chloramphenicol resistance gene (*cat*) (P*_comS_*-*cat* fusion). In absence of XIP_Sth_, native ComR does not dimerize and is unable to bind P*_comS_* (chloramphenicol-sensitive). ComR constitutive mutants will trigger *cat* expression in absence of XIP, conferring resistance to chloramphenicol. *B*, maximum specific luciferase activity (% RLU OD_600_^−1^) of P*_comS_* activation in reporter strains carrying the constitutive ComR_Sth_ mutants in absence or presence of XIP_Sth_ (1 µM) (light or dark gray, respectively). Mutants are grouped according to observed mutations and their positioning in the 3D structure of Apo-ComR_Sth_. Normalization is performed by using as reference the reporter strain producing ComR_Sth_ WT in presence of XIP_Sth_. Experimental values represent the mean ± SD of at least two independent replicates.

**
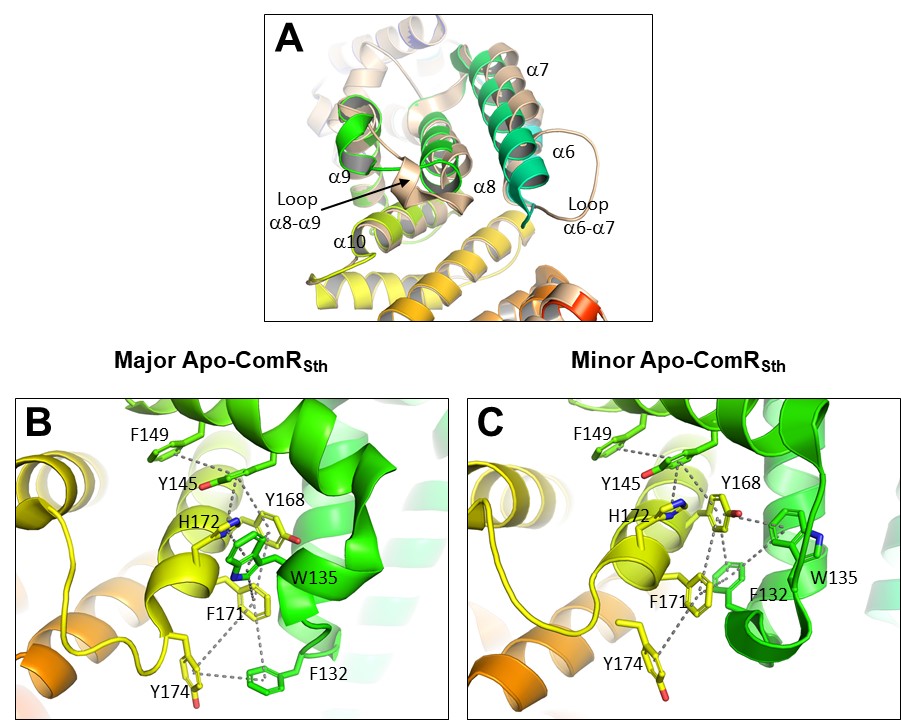
**

**Fig. S4. Remodeling of loop α8-α9 and aromatic-aromatic interactions in apo-ComR_Sth_.** *A*, reorganization of TPR domains in apo-ComR. The major form (colored by spectrum; PDB ID 5JUF (33)) and a minor form (beige; Chain A, PDB ID 6QER (34)) of apo-ComR_Sth_ are superimposed. The remodeling of loop α8-α9 is indicated by an arrow. *B* and *C*, detailed view of F171-Y174 aromatic-aromatic interactions in the major form (colored by spectrum; PDB ID 5JUF (33)) (*B*) and a minor form (colored by spectrum; Chain A, PDB ID 6QER (34)) (*C*) of apo-ComR_Sth_. The network of predicted aromatic-aromatic interactions is modified by the repositioning of F132 and W135 from loop α8-α9. Key residues from helix α9 (Y145 and F149) and helix α10 (Y168, F171, H172 and Y174) are indicated.

**
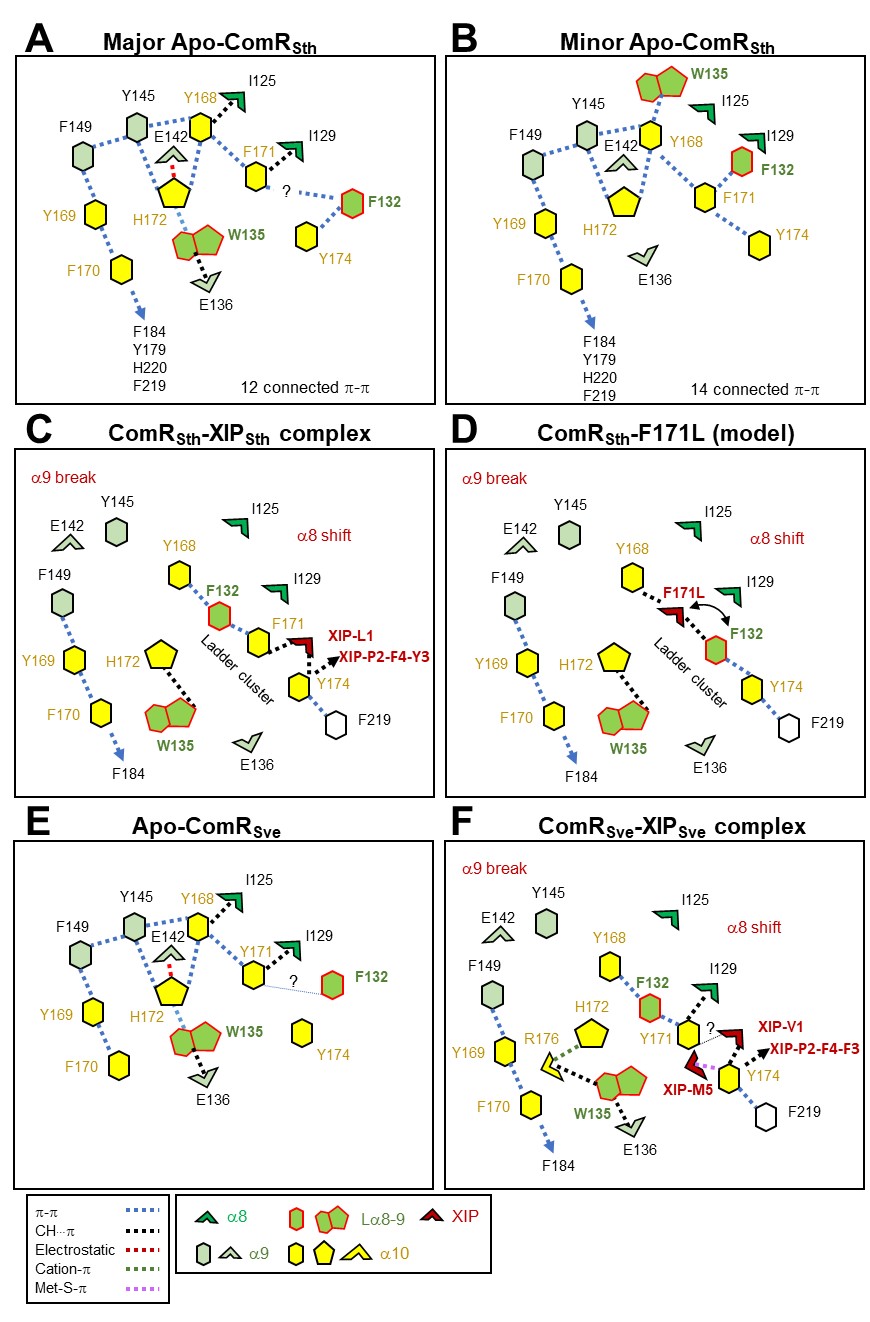
**

**Fig. S5. Topology of the network of aromatic-aromatic interactions.** *A-F*, mapping of interactions in major apo-ComR_Sth_ (PDB ID 5JUF (33)) (*A*), minor apo-ComR_Sth_ (Chain A, PDB ID 6QER (34)) (*B*), ComR_Sth_·XIP_Sth_ complex (PDB ID 5JUB (35)) (*C*), hypothetical model of ComR_Sth_-F171L mutant (*D*), apo-ComR_Sve_ (PDB ID 6HU8 (36)) (*E*), and ComR_Sve_·XIP_Sve_ complex (PDB ID 6HUA (37)) (*F*). Aromatic-aromatic (π-π) (blue), relevant carbon-aromatic (CH^…^π) (black), electrostatic (red), and relevant methionine-sulfur-aromatic (Met-S-π) (fuchsia) interactions were mapped using Arpeggio (http://biosig.unimelb.edu.au/arpeggioweb/) (52). Residues F132 and W135 from loop α8-α9 are surrounded in red to highlight their repositioning in the different aromatic-aromatic networks. The question mark indicates potential F171-F132 (in *A*) or Y171-XIP-L1 (in *F*) interactions slightly above the distance cut-off value.

**Supplemental movies**

**Movie S1**. **TPR-domain remodeling of Apo-ComR_Sth_ upon XIP_Sth_ binding**. Conformational change between apo-ComR_Sth_ (PDB ID 5JUF (33)) and ComR_Sth_**·**XIP_Sth_ complex (PDB ID 5JUB (35)). The local remodeling of key aromatic-aromatic interactions resulting from ComR-F171-Y174/XIP-L1 interactions is highlighted. Shown residues in this conformational change are: F171 (light green spheres), Y174 (dark green spheres), F132 (orange spheres), H172 (cyan spheres), W135 (blue spheres), and XIP-L1 (fuchsia spheres). Shown residues involved in other interactions are: D146-E147 (blue sticks)/R35 (lilac sticks) and E117-E118 (orange sticks)/R39-R51 (lilac sticks) for HTH-domain sequestration, K100 (red sticks) for anchoring XIP-L8, R92 (red spheres) for XIP selectivity, and K87 (yellow spheres) for dimerization.

**Movie S2**. **TPR-domain remodeling of Apo-ComR_Sve_ upon XIP_Sve_ binding**. Conformational change between apo-ComR_Sve_ (PDB ID 6HU8 (36)) and ComR_Sve_**·**XIP_Sve_ complex (PDB ID 6HUA (37)). The local remodeling of key aromatic-aromatic interactions resulting from the interactions between ComR-Y171-Y174 and XIP-V1-M5 are highlighted. Shown residues in this conformational change are: Y171 (light green spheres), Y174 (dark green spheres), F132 (orange spheres), H172 (cyan spheres), W135 (blue spheres), and XIP-V1-M5 (fuchsia spheres).

**Supplemental tables**

**Table S1. Bacterial strains and plasmids used in this study**

|  | **Genotype/description** | **Characteristic** *^a^* | **Reference/source** |
| --- | --- | --- | --- |
| **Strain** | | | |
| ***Escherichia coli*** | | | |
| *E. coli* TOP10 | F- *mcrA* Δ(*mrr-hsdRMS mcrBC*) φ80*lacZ*Δ*M15* Δ*lacΧ74 recA1 araD139* Δ(*ara-leu*) 7697 *galU galK rpsL* (Str^r^) *endA1 nupG λ-* |  | Invitrogen, CA |
| ***Streptococcus thermophilus*** | | | |
| LMD-9 | WT |  | ATCC*^b^* |
| LF121 | LMD‐9 (*blpD*‐*blpX*)::P*_comS_*‐*luxAB* |  | (19) |
| LF134 | LF121 Δ*comS*::P*_32_*‐*cat* | Cm^r^ | (19) |
| LL30 | LF121 Δ*comS*::*comS-11aa* (Δ2-13 aa) | Cm^r^ | This work |
| LL31 | LF121 Δ*comS*::*cat* | Cm^r^ when P*_comS_* is induced by XIP | This work |
| LL32 | LF121 Δ*comS*::*comS-24aa-A5M* | Cm^r^ | This work |
| LL33 | LF121 Δ*comS*::*comS-24aa-L1V-A5M* | Cm^r^ | This work |
| LL34 | LF121 Δ*comS*::P*_comS_*‐*cat,* P*_comS_-comS-11aa* | Cm^r^ when P*_comS_* is induced | This work |
| LF153 | LF121 *comR::comR*-*F171A-Y174A*, Δ*comS*::P*_32_‐cat* | Cm^r^ | (29) |
| LL40 | LF121 *comR::comR*-*F171A*, Δ*comS*::P*_32_‐cat* | Cm^r^ | This work |
| LL41 | LF121 *comR::comR*-*F171L*, Δ*comS*::P*_32_‐cat* | Cm^r^ | This work |
| LL42 | LF121 *comR::comR*-*Y174A*, Δ*comS*::P*_32_‐cat* | Cm^r^ | This work |
| LL43 | LF121 *comR::comR*-*Y174L*, Δ*comS*::P*_32_‐cat* | Cm^r^ | This work |
| **Plasmid** |  |  |  |
| pBADcomRSth-strep | pBADHisA derivative containing the translation fusion P*_ara_*BAD-*comR_Sth_*‐*strep* | Ap^r^ | (19) |
| pGhost9-core | pGhost9 vector derivative forming multimers in presence of the site-specific recombinase TnpI of Tn*4430* | Ery^r^, Ts | Lab. collection |

*^a^* Cm^r^, Ery^r^ and Ap^r^ indicate resistance to chloramphenicol, erythromycin and ampicillin, respectively. Ts, thermosensitive; unstable at 37 °C.

*^b^* ATCC, American Type Culture Collection, Rockville, MD.

**Table S2. Primers used in this study**

| **Oligonucleotide number (#) and name** | | **Sequence (5’‐ 3’) and description** *^a^* | | **Reference** |
| --- | --- | --- | --- | --- |
| **Construction of *S. thermophilus* strains** | | | | |
| 1 | Fw.I.comRSth | TTGAACTTAAAAGACAGCATTGG |  | This work |
| 2 | Rv.PcomSSth+TTG ComS | CAAAATATAACTCCTTTTAACTATTATTTATATTTC | Primers to produce the P*_comS_-comS-11aa* | This work |
| 3 | Fw.ComSSth 11Aa Cterm | GAAATATAAATAATAGTTAAAAGGAGTTATATTTTGATTGCTATCTTGCCTTATTTTGC |  | This work |
| 4 | Rv.DNhypProtComs-lox66 | ataacttcgtatagcatacattatacgaacggtaCATGTATAATAAAAGGGGAAGATAG |  | This work |
| 5 | Fw.DNhypProtComs-lox71 | ataacttcgtataatgtatgctatacgaacggtaGGTTAGGGATGGTGGTATTCAAC |  | This work |
| 6 | Uplox66 | TAAGGAAGATAAATCCCATAAGG | To amplify the P_32_-*cat* cassette | (18) |
| 7 | DNlox71 | TTCACGTTACTAAAGGGAATGTA |  | (18) |
| 8 | Rv.Pre23aaComS Sth | GATAGCAATAAGTAGTGAAAATAGTAC | To include *comS-24aa* variants in strains LL32 and LL33 | This work |
| 9 | Fw.XIP A5M-23aa Sth | CTATTTTCACTACTTATTGCTATCTTGCCTTATTTT**ATG**GGATGTCTTTAATAAGGAGCCATCATGCCAAAATGTC | To include *comS-24aa-A5M* in strain LL32 | This work |
| 10 | Fw.IE1-23aa Sth | CTATTTTCACTACTTATTGCTATC**GTT**CCATACTTC**ATG**GGGTGTCTCTAATAAGGAGCCATCATGCCAAAATGTC | To include *comS-24 aa-L1V-A5M* in strain LL33 | This work |
| 11 | PcomSSth-cat transl.Fw | GTTAAAAGGAGTTATATTTTGaactttaataaaattgatttagacaattg | To create translational fusion P*_comS_-cat* | This work |
| 12 | PcomSSth-cat transl.Rv | gtctaaatcaattttattaaagttCAAAATATAACTCCTTTTAACTATTAttt |  | This work |
| 13 | Fw.hypprotDNcomR | GCAGTTGAGGTTTGAAACTCAG |  | This work |
| 14 | Rv.hypprotDNcomR | CTGAGTTTCAAACCTCAACTGC | To amplify P*_comS_-comS-11aa* | This work |
| 15 | Fw.lox71-EndComRSth | tacattccctttagtaacgtgaaGTATAAAGGCAGGAAAATTGGCAG |  | This work |
| 16 | Fw.ComRSth-F171A | GTCATTGACTATTATTTC**gcT**CATCTTTATGGGAGAAAACAG | To create the ComR-F171A mutant | This work |
| 17 | Rv.ComRSth-F171A | CTGTTTTCTCCCATAAAGATG**Agc**GAAATAATAGTCAATGAC |  | This work |
| 18 | Fw.ComRSth-F171L | GTCATTGACTATTATTTC**TTa**CATCTTTATGGGAGAAAACAG | To create the ComR-F171L mutant | This work |
| 19 | Rv.ComRSth-F171L | CTGTTTTCTCCCATAAAGATG**tAA**GAAATAATAGTCAATGAC |  | This work |
| 20 | Fw.ComRSth-Y174A | GACTATTATTTCTTTCATCTT**gcT**GGGAGAAAACAGTATGAC | To create the ComR-Y174A mutant | This work |
| 21 | Rv.ComRSth-Y174A | GTCATACTGTTTTCTCCC**Agc**AAGATGAAAGAAATAATAGTC |  | This work |
| 22 | Fw.ComRSth-Y174L | GACTATTATTTCTTTCATCTT**Tta**GGGAGAAAACAGTATGAC | To create the ComR-Y174L mutant | This work |
| 23 | Rv.ComRSth-Y174L | GTCATACTGTTTTCTCCC**taA**AAGATGAAAGAAATAATAGTC |  | This work |
| 24 | UpIntComRmut-Fw | TTCTGTTTTAGGAACGATTTTGCTTACAGTTGC | External primers to construct LMD-9 derivative strains | (19) |
| 25 | DNIntComRmut_Rv | AAATCATCAATAATAGC |  | (19) |
| 26 | Fw.extComRSlocus.UP | GCTATTTTTGGAAGAAAAATCAAACAAGC | Confirmation of transformants | (29) |
| 27 | Rv.extComRSlocus.DN | TCATGGAATTTCACCTCAATTTCTTGCTAAC |  | (29) |
| **Primers used to generate the *comR* mutant library** | | | | |
| 28 | Pre comR Sth Fw | AAAGTTTTGATATAAAGGAGATTCTCTTG | Primers for *comR* random mutagenesis | This work |
| 29 | Post comR Sth Rv | ACATTTATGTCACCACCATTTCTA |  | This work |
| 30 | Pre comR Sth.Rv | CAAGAGAATCTCCTTTATATCAAAACTTT | Internal primers for overlapping PCR with *comR* sequences | This work |
| 31 | Post comR Sth.Fw | TAGAAATGGTGGTGACATAAATGT |  | This work |
| **Primers used to generate the library of 11-aa peptide variants** | | | | |
| 32 | Fw.DegeneratedComS11Aa | GTTAAAAGGAGTTATATTTTGATTGCTATC***BTNCCNTWYTTYRYGRKNTRYYWY***TAATAAGGAGCCATCATGCC |  | This work |
| 33 | Rv.ComS11Aa | GATAGCAATCAAAATATAACTCCTTTTAAC |  | This work |
| **Primers used for EMSA experiments** | | | | |
| 34 | Cy3-Fw.ComSboxSth.com. direct | ATAGAAATGGTGGTGACATAAATGTCACTATTTTTTTTAG | Probe Cy3‐ComR box of P*_comS_* from strain LMD‐9 | (29) |
| 35 | Rv.ComSboxSth.com.direct | CTAAAAAAAATAGTGACATTTATGTCACCACCATTTCTAT |  | (29) |

*^a^* Mutant codon introduced in primers is in bold and underlined, and semi-degenerated sequence is indicated in bold and italics.

**Table S3. Synthetic peptides used in this study**

| **Peptide name** | **Sequence** *^a^* | **Reference/source** | |
| --- | --- | --- | --- |
| **Octa or heptapeptides based on *S. thermophilus* pheromone** | | | |
| XIP_Sth_ WT | LPYFAGCL |  | (19) |
| XIP_Sth_-A5T | LPYFTGCL |  | This work |
| XIP_Sth_-A5V | LPYFVGCL |  | This work |
| XIP_Sth_-A5I | LPYFIGCL |  | This work |
| XIP_Sth_-A5M | LPYFMGCL |  | This work |
| XIP_Sth_-L1V-A5M | VPYFMGCL |  | This work |
| XIP_Sth_-L1A | APYFAGCL |  | This work |
| XIP_Sth_-L1A-A5M | APYFMGCL |  | This work |
| XIP_Sth_-Δ1 | PYFAGCL |  | This work |
| XIP_Sth_-Δ1-A5M | PYFMGCL |  | This work |
| **Octa or heptapeptides based on *S. vestibularis* pheromone** | | | |
| XIP_Sve_ WT | VPFFMIYY |  | (19) |
| XIP_Sve_-V1A | APFFMIYY |  | This work |
| XIP_Sve_-M5A | VPFFAIYY |  | This work |
| XIP_Sve_-Δ1 | PFFMIYY |  | This work |
| **Peptides used for FP assays** | | | |
| FITC-XIP_Sth_ WT | *FITC-Ahx-I*LPYFAGCL |  | (30) |
| FITC-XIP-A5M | *FITC-Ahx-I*LPYFMGCL |  | This work |
| **Peptides used for AFM assays** | | | |
| polyG-XIP_Sth_ | *GGGSGGG*LPYFAGCL |  | This work |
| polyG-XIP_Sth_-A5M | *GGGSGGG*LPYFMGCL |  | This work |
| polyG-XIP_Sve_ | *GGGSGGG*VPFFMIYY |  | This work |

*^a^* Substituted residues regarding the wild-type XIP sequence are underlined. Additional residues to the minimal 8-aa XIP and fluorescent dyes are indicated in italics. Ahx, aminohexanoic acid

**REFERENCES**

18. Fontaine, L., Boutry, C., de Frahan, M. H., Delplace, B., Fremaux, C., Horvath, P., Boyaval, P., and Hols, P. (2010) A novel pheromone quorum-sensing system controls the development of natural competence in *Streptococcus thermophilus* and *Streptococcus salivarius*. *J. Bacteriol.* **192,** 1444-1454

19. Fontaine, L., Goffin, P., Dubout, H., Delplace, B., Baulard, A., Lecat-Guillet, N., Chambellon, E., Gardan, R., and Hols, P. (2013) Mechanism of competence activation by the ComRS signalling system in streptococci. *Mol. Microbiol.* **87,** 1113-1132

29. Talagas, A., Fontaine, L., Ledesma-Garcia, L., Mignolet, J., Li de la Sierra-Gallay, Lazar, N., Aumont-Nicaise, M., Federle, M. J., Prehna, G., Hols, P., and Nessler, S. (2016) Structural Insights into Streptococcal Competence Regulation by the Cell-to-Cell Communication System ComRS. *PLoS. Pathog.* **12,** e1005980

30. Ledesma-Garcia, L., Thuillier, J., Guzman-Espinola, A., Ensinck, I., Li de la Sierra-Gallay, Lazar, N., Aumont-Nicaise, M., Mignolet, J., Soumillion, P., Nessler, S., and Hols, P. (2020) Molecular dissection of pheromone selectivity in the competence signaling system ComRS of streptococci. *Proc. Natl. Acad. Sci. U. S. A* **117,** 7745-7754

33. Talagas, A., Fontaine, L., Ledesma-Garcia, L., Li de la Sierra-Gallay, I., Hols, P., and Nessler, S. (2016) Crystal structure of the apo form of ComR from *S. thermophilus*. Protein Data Bank. 5JUF

34. Thuillier, J. and Nessler, S. (2019) Apo Form of ComR from *S. thermophilus* in space group C2. Protein Data Bank. 6QER

35. Talagas, A., Fontaine, L., Ledesma-Garcia, L., Li de la Sierra-Gallay, I., Hols, P., and Nessler, S. (2016) Crystal structure of ComR from *S. thermophilus* in complex with DNA and its signalling peptide ComS. Protein Data Bank. 5JUB

36. Nessler, S., Thuillier, J., Ledesma-Garcia, L., and Hols, P. (2018) Apo form of the competence regulator ComR from *Streptococcus vestibularis*. Protein Data Bank. 6HU8

37. Nessler, S., Thuillier, J., Ledesma-Garcia, L., and Hols, P. (2018) The competence regulator ComR from *Streptococcus vestibularis* in complex with its cognate signaling peptide XIP. Protein Data Bank. 6HUA

52. Jubb, H. C., Higueruelo, A. P., Ochoa-Montano, B., Pitt, W. R., Ascher, D. B., and Blundell, T. L. (2017) Arpeggio: A Web Server for Calculating and Visualising Interatomic Interactions in Protein Structures. *J. Mol. Biol.* **429,** 365-371
